# Neurogenetics of developmental dyslexia: from genes to behavior through brain neuroimaging and cognitive and sensorial mechanisms

**DOI:** 10.1038/tp.2016.240

**Published:** 2017-01-03

**Authors:** S Mascheretti, A De Luca, V Trezzi, D Peruzzo, A Nordio, C Marino, F Arrigoni

**Affiliations:** 1 Child Psychopathology Unit, Scientific Institute, IRCCS Eugenio Medea, Bosisio Parini, Italy; 2 Neuroimaging Lab, Scientific Institute, IRCCS Eugenio Medea, Bosisio Parini, Italy; 3 Department of Information Engineering, University of Padova, Padova, Italy; 4 Centre for Addiction and Mental Health, University of Toronto, Toronto, ON, Canada

## Abstract

Developmental dyslexia (DD) is a complex neurodevelopmental deficit characterized by impaired reading acquisition, in spite of adequate neurological and sensorial conditions, educational opportunities and normal intelligence. Despite the successful characterization of DD-susceptibility genes, we are far from understanding the molecular etiological pathways underlying the development of reading (dis)ability. By focusing mainly on clinical phenotypes, the molecular genetics approach has yielded mixed results. More optimally reduced measures of functioning, that is, intermediate phenotypes (IPs), represent a target for researching disease-associated genetic variants and for elucidating the underlying mechanisms. Imaging data provide a viable IP for complex neurobehavioral disorders and have been extensively used to investigate both morphological, structural and functional brain abnormalities in DD. Performing joint genetic and neuroimaging studies in humans is an emerging strategy to link DD-candidate genes to the brain structure and function. A limited number of studies has already pursued the imaging–genetics integration in DD. However, the results are still not sufficient to unravel the complexity of the reading circuit due to heterogeneous study design and data processing. Here, we propose an interdisciplinary, multilevel, imaging–genetic approach to disentangle the pathways from genes to behavior. As the presence of putative functional genetic variants has been provided and as genetic associations with specific cognitive/sensorial mechanisms have been reported, new hypothesis-driven imaging–genetic studies must gain momentum. This approach would lead to the optimization of diagnostic criteria and to the early identification of ‘biologically at-risk’ children, supporting the definition of adequate and well-timed prevention strategies and the implementation of novel, specific remediation approach.

## Introduction

Reading is a cognitive skill unique to humans and crucial for living in the modern society. To be a successful reader, one must rapidly integrate a vast circuit of brain areas with both great accuracy and remarkable speed. This ‘reading circuit’ is composed of neural systems that support language as well as visual and orthographic processes, working memory, attention, motor functions and higher-level comprehension and cognition.^1^ Nevertheless, for about 5 to 12% of the population, learning to read is extremely difficult.^2^ These individuals are affected by a complex neurodevelopmental disorder called developmental dyslexia (DD), which represents the most common learning disability among school-aged children and across languages. DD is a lifelong impairment^2^ characterized by impaired reading acquisition in spite of adequate neurological and sensorial conditions, educational opportunities and normal intelligence.^3^ This difficulty in reading is often associated with undesirable outcomes for children as well as with social impact and economic burden.^2^

Although the field is immature, the role of genetics in DD is rapidly growing and much has been learned regarding the possible downstream effects of DD risk genes on the brain structure, function and circuitry. Similarly, cognitive and psychophysic studies have provided initial evidence about the usefulness of testing well-identified cognitive and sensorial deficits associated with and causative of DD to pursue the biological and genetic components of this disorder. Following the increasing findings provided by molecular genetic, cognitive and imaging–genetic studies of DD, this review aims to propose an interdisciplinary, multilevel, imaging–genetic approach to disentangle the pathways from genes to behavior. An interdisciplinary integration of particular cognitive/sensorial, selective genetic, and imaging data, will provide a critically important bridge for ‘connecting the dots’ between genes, cells, circuits, neurocognition, functional impairment and personalized treatment selection, and will pave the way for new candidate gene–candidate phenotype imaging association studies.^4^

## Genetics of DD

Following earlier descriptions of strong familial aggregation of the disorder,^5^ substantial heritability typical of a complex trait has been reported^6^ with estimates across DD and DD-related quantitative phenotypes ranging from 0.18 to 0.72.^7^ Since the early 1980s, at least nine DD risk loci termed *DYX1*–*DYX9* on eight different chromosomes have been mapped (that is, 1p36-p34, 2p16-p15, 3p12-q13, 6p22 and 6q13-16.2, 11p15.5, 15q21.3, 18p11.2 and Xq27.3) and the involvement of several genes spanning these regions in the etiology of DD has been reported (that is, *DYX1C1*, *DCDC2*, *KIAA0319*, *C2ORF3*, *MRPL19*, *ROBO1*, *FAM176A*, *NRSN1*, *KIAA0319L* and *FMR1*).^8, 9, 10, 11, 12, 13^ Apart from these DYX loci, other genes implicated in other disorders, before being examined for DD, have also been associated with reading (dis)ability, that is, *FOXP2*, *CNTNAP2*, *DOCK4* and *GTF2I* on chromosome 7,^14, 15, 16, 17^ *GRIN2B* and *SLC2A3* on chromosome 12,^18, 19, 20^ *ATP2C2* and *CMIP* on chromosome 16,^15, 21^ *PCNT*, *DIP2A*, *S100B* and *PRMT2* on chromosome 21.^21, 22, 23^ Recent genome-wide association and sequencing studies further strengthened the role of previously identified DD-candidate genes^22, 24, 25^ and identified novel associations with markers spanning new chromosomal regions.^12, 22, 24, 26, 27, 28, 29, 30^ Among all these genes, nine DD-candidate genes have been replicated in at least one independent sample: *DYX1C1*, *DCDC2*, *KIAA0319*, *C2ORF3*, *MRPL19*, *ROBO1*, *GRIN2B*, *FOXP2* and *CNTNAP2*.^8, 9, 10, 11, 12, 18, 20,,31^ Interestingly, initial evidence has been provided of the presence of putative functional genetic variants influencing the expression of some of the above-described DD-candidate genes. A functional effect of two single-nucleotide polymorphisms (SNPs) in *DYX1C1*, rs3743205 (-3G→A) and rs57809907 (1249C→T), has been hypothesized on the basis of bioinformatics predictions.^32^ In particular, the -3G→A SNP is located in the binding sequence of the transcription factors Elk-1, HSTF and TFII-I, and affects the Kozak sequence, which has a major role in the translation process. The coding 1249C→T-SNP truncates the protein and thus likely disrupts its functionality.^32^ These two *DYX1C1* variants have been associated with DD and DD-related phenotypes,^32, 33, 34^ although opposite patterns of effects^35, 36, 37, 38, 39, 40, 41, 42^ and negative findings^43^ have also been observed. A three-SNP risk haplotype spanning across *TTRAP*, *THEM2* and *KIAA0319* genes, has been described, that is, rs4504469, rs2038137 and rs2143340.^44^ This risk haplotype is associated with 40% lower levels of the expression, splicing or transcript stability of any of the *KIAA0319*, *TTRAP* or *THEM2* genes as compared with the non-risk haplotype.^44^ Furthermore, it has been shown to associate with DD in three independent clinical samples,^44, 45, 46, 47^ as well as in two large unselected samples.^48, 49^ Further characterization of *KIAA0319* has led to the identification of a marker in the risk haplotype, that is, rs9461045, found to be strongly associated with DD and to influence gene expression, possibly due to the alteration of the binding site to transcriptional silencer OCT-1 by luciferase-based assays.^47^ Interestingly, a 168-base pair purine-rich region in the intron 2 of the *DCDC2* gene harboring a highly polymorphic, short-tandem repeat (BV677278) has been reported.^50^ This non-coding region might serve as a regulatory node as it contains 131 putative transcription factor binding sites, is rather conserved across species and has the capacity of enhancing activity, as BV677278 changes the reporter gene expression from the *DCDC2* promoter in an allele-specific manner.^51^ Although more work is needed to confirm it, Powers *et al.*^52^ recently identified the BV677278-binding protein as the transcription factor ETV6, confirmed BV677278 as a regulatory element and proposed ‘regulatory element associated with dyslexia 1’ (READ1) as a new name. As such, READ1 could substantially act as a modifier of *DCDC2* gene expression. A naturally occurring deletion in intron 2 of the *DCDC2* gene (hereafter, *DCDC2*d), encompassing READ1, has been associated with DD and DD-related phenotypes,^34, 37, 46, 50, 53, 54^ although negative findings have also been reported.^41, 55^ In accordance with works showing that cognitive traits can be useful in the search for the susceptibility genes of neurodevelopmental disorders,^56^ two recent psychophysical studies showed that *DCDC2*d specifically influences the inter-individual variation in motion perception both in children with DD^57, 58^ and in normal readers.^58^ Finally, one of the most informative reports of a specific loss of *CNTNAP2* function has come from a study of an old-order Amish population in which 13 probands were found to carry the same homozygous point mutation within *CNTNAP2*, that is, 3709delG.^59^ This change introduced a premature stop codon (I1253X) predicted to produce a non-functional protein.^59, 60^

Recent evidence has shown that DD-susceptibility genes affect neuronal migration, neurite outgrowth, cortical morphogenesis and ciliary structure and function.^25, 27, 50, 61, 62, 63, 64, 65, 66, 67, 68, 69, 70, 71, 72, 73, 74, 75, 76, 77, 78, 79, 80, 81, 82^ In particular, *ROBO1* is known to be an axon guidance receptor regulating the connections between brain hemispheres.^25, 61, 62, 63^ The protein encoded by *DYX1C1* has been linked to neuronal migration, estrogen receptor transport and cilia structure and functions.^64, 65, 66, 71, 74, 78, 81^ Animal studies showed that *in utero* RNAi of *DYX1C1* is related to deficits in both RAP, spatial working memory performance, as well as learning and memory performance.^9, 83^ The expression pattern of *KIAA0319* in the developing neocortex is consistent with its hypothesized role in neuronal migration, and recent bioinformatics analysis has suggested its involvement in ciliary functions.^69, 70, 72, 75, 79, 80, 84^ The embryonic RNAi of *KIAA0319* expression results in RAP and spatial learning deficits.^9, 85^ The *DCDC2* gene encodes a protein with two DCX domains which are essential for neurite outgrowth and neuronal migration and it is involved in ciliary functions.^27, 50, 67, 81, 86^ *DCDC2* knockout mice show impairments in visuospatial memory, visual discrimination and long-term memory, auditory processing, working memory and reference memory.^9, 87, 88^ Similarly, animal studies have shown that the *Glun2b* subunit is required for neuronal pattern formation in general and for channel function and formation of dendritic spines in hippocampal pyramidal cells in particular.^68, 89, 90, 91^ Recently, *DCDC2* knockout mice were shown to have increased excitability and decreased temporal precision in action potential firing,^92^ as well as increased functional excitator connectivity between layer 4 lateral connections in the somatosensory neocortex^93^ mediated by subunit *Grin2B*. Focused functional investigations of cellular and mouse models uncovered connections between *FOXP2* and neurite outgrowth.^73, 77^*FOXP2* was first implicated in a family segregating a severe form of dyspraxia of speech, designated the KE family.^94, 95^ Since its original identification, many studies reported that rare variants disrupting one copy of *FOXP2* cause language-based learning (dis)abilities-related impairment.^31^ Mice carrying mutant *Foxp2* exhibit abnormal ultrasonic vocalizations as well as other disorders including developmental delay, deficits in motor-skill learning and impairments in auditory–motor association learning.^96, 97, 98, 99, 100, 101^ FOXP2 encodes a forkhead domain transcription factor expressed in several brain structures^102^ and modulates the DNA transcription at numerous loci throughout the genome. *CNTNAP2* is one of its gene targets^103^ and it has recently been implicated in a broad range of phenotypes including autism spectrum disorder, schizophrenia, intellectual disability, DD and language impairment.^104^ *CNTNAP2* encodes a cell-surface neurexin protein, that is, CASPR2, implicated in neuronal connectivity at the cellular and network level, interneuron development/function, synaptic organization and activity and migration of neurons in the developing brain.^104^ Recently, a genetic knockout of the rodent homolog *Cntnap2* has been associated with poor social interactions, behavioral perseveration and reduced vocalizations, as well as with delayed learning and cross-modal integration.^105, 106^ In contrast, little is known about the *C2ORF3* and *MRPL19* candidate genes. *C2ORF3* protein is suggested to have a potential function in ribosomal RNA (rRNA) processing,^107^ and, as for *MRPL19*, is highly expressed in all areas of fetal and adult brain.^108^Furthermore, their expression was strongly correlated with *DYX1C1*, *ROBO1*, *DCDC2* and *KIAA0319* across different brain regions.^108^ All these findings depict DD as a disorder at the mild end of the spectrum of a number of pathways producing developmental disturbances in neuronal positioning and axonal outgrowth,^109^ consistent with the neuroanatomical findings of focal architectonic dysplasia and neuronal ectopias in the brains of people with DD.^110^

## Imaging in DD

**Figure 1:**
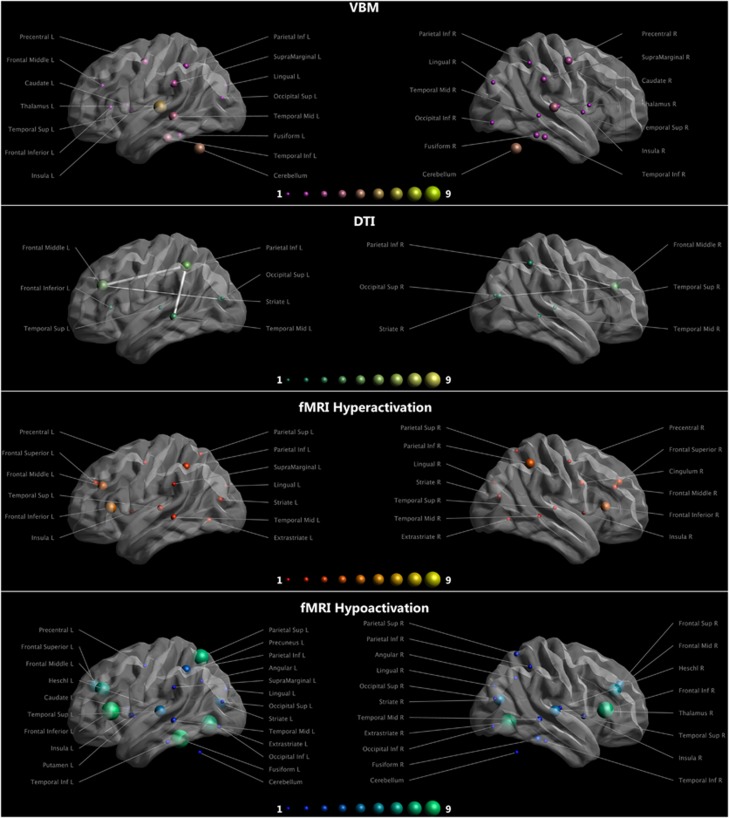
Rows show the findings obtained with structural and functional MR techniques in DD subjects. The size and the color of the spheres reflect the amount of papers reporting differences in the specified area. Longitudinal fascicoli and arcuate fasciculus are shown as edges. fMRI findings are not divided by task. Task specific findings are available in Supplementary Tables 1 and 2. DD, developmental dyslexia; fMRI, functional magnetic resonance imaging. Figure was created with ExploreDTI (http://exploredti.com). DTI, diffusion tensor imaging; VBM, voxel-based morphometry.

Postmortem studies in DD patients showed reduced left–right asymmetry of the planum temporale,^111^ as well as neuronal ectopias and architectonic dysplasias in the left perisylvian regions.^110^ More recently, magnetic resonance imaging (MRI) has been extensively used to investigate both morphological, structural and functional brain abnormalities in DD patients (Figure 1). Being noninvasive and allowing *in vivo* studies, MRI is a unique and valuable tool for disentangling tissue modifications and functional (re)organization in developmental disorders like DD. Among different MRI-based techniques, voxel-based morphometry (VBM) is used to quantify gray and white matter (GM and WM, respectively) volumes, while diffusion tensor imaging (DTI), which probes water diffusivity in the micron scale, detects alterations in WM structure and indirectly in the architecture of fiber pathways. Finally, functional MRI (fMRI) investigates brain activations during cognitive and sensory tasks, and when at rest.

### VBM analysis

By applying VBM, altered GM density has been identified in several areas, that is, in the left temporal and parietal regions,^112, 113, 114, 115, 116, 117, 118, 119^ bilaterally in the fusiform gyrus, lingual gyrus, temporo-parieto-occipital junction, frontal lobe, planum temporale, inferior temporal cortex, caudate, thalamus and cerebellum,^115, 118, 119, 120, 121, 122, 123, 124, 125, 126^ and in the right parietal lobe.^123, 125^ Moreover, VBM analysis has revealed altered WM density in the bilateral temporal and frontal lobes, in the left cuneus and arcuate fasciculus, and in the right precuneus and cerebellum.^113, 116, 117, 118, 119, 122, 124, 125^

### DTI analysis

Alterations of WM structure have been found in bilateral tracts within the frontal, temporal, occipital and parietal lobes,^124, 127, 128, 129^ in the superior longitudinal fasciculus,^130, 131^ in the left superior corona radiata, in the left centrum semiovale,^132^ in the left inferior frontal gyrus and temporo-parietal WM,^133^ in the left middle and inferior temporal gyri^113^ and in the left arcuate fasciculus.^113, 134^ Moreover, several studies have reported significant differences in the corpus callosum.^135, 136^

### fMRI analysis

fMRI has had an important role in understanding the pathophysiology of DD by analyzing the brain areas activated while performing specific tasks. The brain activations associated with the reading process have been extensively analyzed using fMRI, as well as other reading-related functions, such as phonological processing, integration of letters and speech, visual perception and attention, working memory and acoustic stimuli.^137, 138^ Depending on the task performed during fMRI, several altered activation patterns have been reported.

With reading-related tasks, altered activations were found in the DD subjects in the left hemispheric temporo-parietal regions (Brodman’s areas (BAs) 20, 21, 37, superior and middle temporal gyrus, operculum, supplementary motor area), and in the bilateral frontal and occipital areas (BAs 44 and 45, inferior and middle frontal gyrus, visual areas and extrastriate cortex).^139, 140, 141, 142, 143, 144, 145, 146, 147, 148^

Subjects with DD showed abnormal activity during phonological tasks in the left hemispheric temporal areas (Rolandic operculum, middle and superior temporal gyrus, fusiform gyrus, planum temporale and Wernicke’s area), in bilateral parietal (superior and inferior parietal gyrus, BA40), frontal (BAs 44 and 45, middle and inferior frontal gyrus, precentral gyrus, superior medial gyrus and prefrontal cortex), occipital cortex (middle and superior occipital gyrus, lingual gyrus, calcarine sulcus, BAs 18 and 19, striate cortex), cerebellum, and right hemispheric subcortical structures (putamen, basal ganglia).^149, 150, 151, 152, 153, 154, 155, 156, 157, 158, 159, 160, 161^

During semantic tasks, diffuse activations have been reported in DD subjects in the left hemispheric temporal (BA22, fusiform gyrus, parahippocampal gyrus and middle and superior temporal gyrus) and occipital (V5/MT), as well as bilateral parietal (inferior parietal lobule, supramarginal gyrus), frontal (BAs 44 and 45, precentral gyrus, superior frontal gyrus) cortex, cerebellum and subcortical structures.^162^

Children with DD showed altered activations during auditory tasks in the right temporal areas (middle and superior temporal gyrus, BAs 41 and 42, Heschl gyrus, superior temporal cortex), anterior insular cortex, cingulate cortex, thalamus and cerebellum, in the left occipital (cuneus) and parietal (inferior parietal region, supramarginal gyrus, angular gyrus) regions and in bilateral frontal areas (supplementary motor area, inferior and middle frontal gyrus, precentral gyrus, inferior frontal sulcus, prefrontal cortex).^152, 153, 163, 164, 165, 166, 167, 168, 169^

Working memory-related tasks elicited altered activations in the bilateral parietal (superior parietal cortex, inferior parietal lobule) and frontal (BA46, prefrontal cortex, inferior frontal gyrus) areas in children with DD.^170, 171, 172, 173^

The reduced activation of the primary visual cortex, extrastriatal areas and the V5/MT area during fMRI using visual stimuli,^174, 175, 176^ as well as increased right frontal activation in areas 44 and 45 (ref. 152) have been consistently reported in subjects with DD. Visual spatial tasks elicited altered activation in the right temporal (temporal pole, fusiform gyrus, temporal gyrus, motor/premotor cortex) and frontal (precentral gyrus, frontal gyrus) areas, and in bilateral parietal (intraparietal sulcus, inferior and superior parietal lobes, precuneus), occipital (cuneus, BAs 17–19), subcortical structures (putamen, basal ganglia), anterior cingulate and cerebellum.^157, 166, 177, 178^

Altered activations in bilateral temporal (inferior temporal cortex), parietal, frontal (middle frontal cortex), occipital (striate and extrastriate visual cortex) and cingulate cortex have been reported during attentional tasks in children with DD.^179, 180, 181^

Interestingly, the fMRI activation patterns in response to tasks requiring the processing of several demands (visuospatial, orthographic, phonologic and semantic) showed that subjects with DD tend to process using the visuospatial areas instead of the normal language processing areas.^150, 169^

Results of imaging studies on pre-reading children at risk for DD are in agreement with results found for children with DD,^182, 183, 184, 185^ suggesting that neural alterations in DD predate reading onset, reflect the differential developmental trajectory of reading brain networks and may serve as early biomarkers of risk for DD.

Given the heterogeneity of imaging modalities and findings, it is difficult to summarize MR results into a unifying perspective (Figure 1). According to previous findings showing a consistent link between reading and both subcortical structures and cortical systems, structural techniques (VBM and DTI) identify temporo-parietal and, partially, middle frontal areas as the targets of cerebral derangement that may occur in DD, whereas more anterior and occipital areas seem to be less frequently involved. It is even harder to sum up the findings derived from functional MR studies. In broad terms, a pattern of cerebral hypoactivation seem to prevail over hyperactivity during task-based fMRI. Circuits involving temporo-basal, parietal and frontal lobes are more frequently impaired, without a clear lateralization between the left and right hemispheres.

The details about the study design and results are reported in Supplementary Information 1 and 2.

## Imaging–genetics in DD

Taken together, these findings show how neuroimaging and genetic research have substantially enhanced understanding of the mechanisms underlying atypical reading development. Despite the successful characterization of DD-susceptibility genes, we are far from achieving a comprehensive understanding of the pathways underlying the development of DD.^186^ By focusing mainly on clinical phenotypes, the molecular genetics approach has yielded mixed results,^187^ including negative findings for the DD-candidate genes.^42, 188, 189, 190^ This could be ascribed to at least three possible sources: (1) as complex traits are substantially polygenic, with each variant having a small effect, larger sample sizes are needed,^191^ (2) the pathway from genes to phenotypes is not straightforward (see for example, ‘the missing heritability problem’)^192^ and can be influenced by incomplete linkage disequilibrium between causal variants and genotyped SNPs,^193^ environmental, gene-by-gene and gene-by-environment effects,^2, 186^ (3) it is unlikely that a single model connects all the DD-candidate genes and their corresponding proteins at the molecular level, therefore several etiological cascades involved in neuronal migration and neurite outgrowth contributing to DD likely exist.^194^

An alternative approach is to focus on the phenotypes thought to reflect lower-level processes, hypothesizing that individual differences in the areas responsible for reading acquisition might be important end points, better reflective of the underlying biology and more tractable to genetic mapping than behavioral phenotypes.^56, 195^ In addition, the brain is the most complex of all organs, and behavior is not merely the sum of the phenotypic output of complex interactions within and between endogenous and exogenous environments during development. Therefore, more optimally reduced measures of functioning (hereafter, intermediate phenotypes—IPs) should be more useful than behavioral ‘macros’ in studies pursuing the biological and genetic components of neurodevelopmental disorders.^196^ Genetic determination of an IP will likely be less complex than determination of the related behavioral/clinical phenotype, as the latter incorporates multiple neural systems and is influenced by multiple genes and environmental etiologic variables.^186^ Even if concerns have been raised about how to interpret the relationship between IPs and psychiatric disorders,^197^ such use of IPs has had a crucial role in improving the knowledge of the gene to phenotype gap in other neurodevelopmental disorders (for example, schizophrenia—SKZ, autism spectrum disorder).^195^

Imaging data provide a viable IP for complex neurobehavioral disorders like DD, reducing the inherent complexity of brain functioning and of the intricate clinical outcome of these disorders.^56, 196, 197, 198^ Performing joint genetic and neuroimaging studies in humans, where the association between genotypes and brain phenotypes can be tested, is an emerging strategy to link DD-candidate genes to brain structure and function. To date, imaging–genetic studies, including both structural and functional imaging, have focused on at least one of the above-described DD-candidate genes and on the proposed functional variants spanning them (Table 1).^17, 199, 200, 201, 202, 203, 204, 205, 206, 207, 208, 209, 210, 211, 212, 213, 214, 215^ Although some of the studies involving DD-candidate genes have been carried out on populations other than DD (that is, healthy subjects, SKZ), they have been taken into consideration for the purpose of this review, that is, to propose an interdisciplinary, multilevel, imaging–genetic approach to disentangle the pathways from genes to behavior, by focusing on selective, functional genetic variants and particular, well-defined cognitive/sensorial phenotypes. Structural MRI studies have shown that in subjects with SKZ and controls, *DYX1C1* and *KIAA0319* genes are significantly correlated with the inferior and superior cerebellar networks,^201^ with WM volume in the left temporo-parietal region,^203, 204^ and with cortical thickness in the left orbitofrontal region in typically developing children.^208^ A pilot resting-state fMRI study failed to find a significant link between *DYX1C1* markers and functional connectivity of language-related regions in both subjects with SKZ and healthy controls.^202^ Functional MRI studies showed associations between *KIAA0319* and asymmetry in functional activation of the superior temporal sulcus,^205^ and the inter-individual variability in activation of reading-related regions of interest (that is, the right and left anterior inferior parietal lobe)^199^ during reading-related tasks in two independent samples of subjects with DD and normal readers. Moreover, *KIAA0319* was found to influence functional connectivity in language-related regions (that is, a left Broca-superior/inferior parietal network, a left Wernicke-fronto-occipital network and a bilateral Wernicke-fronto-parietal network) in both subjects with SKZ and healthy controls.^202^ In healthy adults, an allelic variation in the *DCDC2* gene has been associated with individual differences in cortical thickness,^204^ and in fiber tracts, which are commonly found altered in neuroimaging studies of reading and DD (that is, the connection of the left medial temporal gyrus with the angular and supramarginal gyri, the superior longitudinal fasciculus and the corpus callosum).^203^ Interestingly, in a sample of subjects with SKZ and controls*, DCDC2* was found to be associated with distributed cortical structural abnormalities in language-related superior prefrontal, temporal and occipital networks,^201^ and with inter-individual variations in functional connectivity in a Broca-medial parietal network.^202^ Furthermore, in healthy adults, *DCDC2*d has been associated with altered GM volumes in reading/language-related brain regions especially in the left hemisphere,^200^ and with both common and unique alterations of WM fiber tracts in subjects with DD.^207^ In an fMRI study, Cope *et al.*^199^ found significant associations between *DCDC2*-READ1 and brain activations in the left antero-inferior parietal lobe and in the right lateral occipital temporal gyrus during reading tasks, and a nominally significant association between *DCDC2*d and activation in the left antero-inferior parietal lobule. Further imaging–genetic studies investigated the effects of *C2Orf3*/*MRPL19* and *GRIN2B* genes upon neuroanatomical structures. By using VBM, Scerri *et al.*^206^ revealed that WM volume in the bilaterally posterior part of the corpus callosum and the cingulum varied depending on one variant in the *C2Orf3*/*MRPL19* region. Finally, in healthy individuals, *GRIN2B* correlated negatively with dorsolateral prefrontal cortex activity during a working-memory-related task.^209^ Imaging–genetics of *FOXP2* and *CNTNAP2* has implicated common genetic variants spanning these genes. Multiple imaging studies of the KE family have found both structural and functional alterations in subjects with dyspraxia of speech and the mutant *FOXP2*.^216, 217, 218, 219^ Even if no evidence for effects of *FOXP2* on variability in brain structures in a sample of >1300 people from the general population have been recently reported,^210^ common variants spanning this gene were associated with altered levels of activation in temporo-parietal and inferior frontal brain areas during both reading and speech listening tasks in DD samples.^17, 205^ *CNTNAP2* has been associated with structural brain connectivity and brain activation in BA7, BA44 and BA21 during a language processing task in healthy individuals.^211, 212^ Moreover, it has been significantly associated with FA in the uncinate fasciculus of subjects with SKZ,^213^ with reduction of GM and WM volume and lower FA in the cerebellum, fusiform gyrus, occipital and frontal cortices,^214^ and with modulation in functional frontal lobar connectivity^215^ in subjects with a diagnosis of autism spectrum disorder.

## Limitations of current imaging–genetic studies

Clearly, neuroimaging is playing a fundamental part in disentangling the role of genetic variants in the etiology of complex cognitive functions like reading. However, the complexity of the ‘reading circuit’ is still far from being completely understood, as revealed by the heterogeneous and sometimes conflicting results of brain MRI studies.

Study design and data processing are important factors increasing complexity and heterogeneity in neuroimaging research. The inclusion of subjects with an unknown genetic profile will likely enhance inter-subject variability, as different DD genes may cause different deficits in different, particular cognitive and sensorial phenotypes (see ‘Genetics of DD’ paragraph). Nevertheless, even if some imaging–genetic studies of DD have been proposed,^17, 199, 200, 201, 202, 203, 204, 205, 206, 207, 208, 209, 210, 211, 212, 213, 214, 215^ the number of these works is still too low to draw definitive conclusions about the role of each DD-candidate gene.

Moreover, it is interesting to note some technical evidence that might limit the integration of these results. Of the 19 aforementioned imaging–genetic studies, 10 have used 1.5T scanners,^199, 203, 204, 205, 206^ eight were performed with 3T scanners^200, 201, 202, 205, 207, 208, 209, 215^ and one acquired with a 4T scanner.^211^ Two of them used similar acquisition protocols and performed VBM to investigate GM,^200, 201^ but their results were only partially overlapping. These different findings may be owing to the different disorders included in the studies (that is, DD and SKZ) and/or to the different analysis pipelines (linear regression versus independent component analysis). Genetic data can be integrated with every parametric map derived from MRI, whether a simple measure of volume, a microstructure-related metric or a measure of chemical properties. Three of the aforementioned studies integrated genetic data in the VBM analysis of WM volume as an attempt to reveal genetically related alterations, limiting the analysis of DTI data to the detection of the major fiber bundles included in altered WM areas.^203, 204, 206^ Nevertheless, DTI analysis can provide parameters that are more specific to WM microstructure than VBM,^220^ including fractional anisotropy (FA) and measures of diffusivity along different spatial axes. These maps can be analyzed similarly to VBM, but may provide additional characterization of the genetic effect at the microstructural level. To date, only three studies have used DTI-derived maps to detect voxel-based WM modifications related to DD-candidate genes.^207, 208, 214^ One of the studies^213^ computed FA maps and tried to perform region-of-interest-based analysis of covariance regression with the SNPs of *CNTNAP2*; however, only one genotype was a significant predictor of FA in the uncinate fasciculus after Bonferroni correction, despite the relatively high number of subjects included in the study (*n*=125). Further studies with rigorous advanced diffusion MRI protocols (that is, high-field magnets, multiple directions and b-values) and populations with a specific genetic characterization are therefore needed. Moreover, more complex diffusion-based techniques, such as NODDI (Neurite Orientation Dispersion and Density Imaging), have recently provided more specific metrics of GM and WM in several applications.^221, 222, 223^ The application of NODDI or other affine techniques might be beneficial to the study of DD, providing additional disentanglement of the connections between genetic variations and structural alterations.

Similar considerations apply to fMRI, where the choices of stimuli and the analysis pipeline are fundamental. To date, functional imaging–genetic studies of DD have investigated the effects of DD-candidate genes only during reading tasks,^199, 205, 209^ irrespective of the deficits each DD gene is likely to produce (see ‘Genetics of DD’ paragraph). Moreover, while task-based fMRI might help investigate the effects of DD-candidate genes on specific brain functions through correlation analysis or linear regressions, resting-state fMRI might offer a more reproducible/reliable approach to the investigation of genetic effects on brain functionality. It is worth noticing that while imaging–genetic studies are at their early stages in DD, they are more popular in the context of other diseaes.^224, 225, 226, 227^ For example, the ADNI (Alzheimer’s Disease Neuroimaging Initiative)^228^ has performed MRI and positron emission tomography acquisitions with genetic profiling in more than 1000 subjects over time. Along with genetic profiling, the success of the initiative is strongly supported by the standardization of multicentric acquisition protocol and processing methods, all factors that are unfortunately still lacking in imaging–genetic studies on DD.

## Toward a new approach

As aforementioned, learning to read requires the accurate, fast and timely integration of different neural systems supporting different cognitive and sensorial processes. Molecular genetic studies have consistently identified DD-candidate genes and provided initial evidence of the presence of putative functional genetic variants influencing gene expression. Recent findings in both animal and humans studies support the role of specific genetic variants on the different cognitive and sensorial processes underlying reading acquisition. Similarly, neuroimaging data can be considered IPs to genetics in identifying the causes of DD.^198^ New studies must therefore gain momentum to understand the function of neuronal migration genes and their relationships with specific cognitive and sensorial vulnerability, and to establish links between such susceptibility variants and neuroanatomical phenotypes. Following a probabilistic and multifactorial etiological model of reading acquisition, the emergence of DD is rooted at multiple levels, and may reflect the global failure of interacting mechanisms, each with degrees of impairment that vary across children.^2, 186, 229, 230, 231, 232^ It is therefore reasonable to predict a low specificity and high heterogeneity of imaging findings, especially when dealing with small sample sizes. Furthermore, according to this model, the fundamental role of genetics in the selection of homogeneous DD subtypes population suitable for imaging investigation appears reasonable. The integration of specific cognitive/sensorial, selective genetic and imaging data can lead to the identification of regions with gene- and cognitive/sensorial-specific effects (that is, only a risk genetic variant alters structure/function in this region tapping specific cognitive/sensorial mechanisms) or with universal effects (that is, all/many-risk gene function in this region). Identifying the dots connecting putative functional genetic variants, neuroanatomical structures and functions, and reading-related cognitive/sensorial pathways, will be important areas for imaging–genetics research in the future and will pave the way for new candidate gene–candidate phenotype imaging association studies.^4^ However, some have argued that neuroimaging studies reporting effects of candidate genes are also at risk for false-positive effects due to small sample sizes, and questions about the statistical power of imaging techniques may be risen.^233, 234^ Some possible strategies could be used to overcome such variability. First, accordingly to what is proposed in this review, an alternative way to avoid false positives is to focus on selective variants with known molecular function and to take into account the increment in effect sizes enabled by careful selection of phenotypes.^235, 236^ By narrowing the search space to genes that are likely to have a role—and whose functions have more chance of being understood—the power of the study is directly increased, as is its practical value for neuroscience and medicine.^235^ The identification of what constitutes a phenotype is crucial as the identification of the phenotype itself. Going beyond classical association studies, where heterogeneous patient groups selected by clinical symptoms are compared with controls, is crucial to identify reliable biomarkers and to guide the diagnosis of neurodevelopmental disorders.^4^ More specific, elementary, straightforward IPs may help to interpret the results of genetic studies of psychiatric diseases,^233^ increasing the statistical power in smaller sample size.^236^ Recent studies on relatively small samples show that using IPs can be very useful for researching susceptibility genes in DD^26, 237, 238^ and for explaining their effects on the phenotypic variance.^35, 57, 58^ Second, there is a growing perception of reproducibility as a fundamental building block in science. Some have argued that small individual studies—when replicated—may lead to useful observations to address the impact of genetic variation on a neural system that is abnormal in a given illness, despite the problem of false-positive findings. An alternative strategy is to recruit large data sets through multicenter studies. Many neuroimaging consortia have been recently established (for example, the ADNI, the functional Brain Imaging Research Network, the Mind Clinical Imaging Consortium, the Enhancing NeuroImaging Genetics through Meta-Analysis consortium, the Pediatric Imaging Neurocognition Genetics study) to expand the promise of imaging–genetic studies and to detect factors that affect the brain that could hardly be detected by single site studies.^12, 235^ However, as some limitations apply (for example, it is difficult to aggregate data from cohorts that are heterogeneous in terms of duration of illness and demographics, spoken languages, ethnic differences in allele frequency), novel, harmonized data analysis and meta-analysis protocols checking for the effects of possible confounders, are crucial to the success of these projects.^235, 239^ Third, it would help to develop an interdisciplinary multilevel approach aimed at defining MRI protocols heavily guided by genetics and cognitive findings. The best outcomes result from cooperation within a multidisciplinary team to address the different levels of investigation underlying such complex neurodevelopmental disorders.^240, 241^ Nonetheless, addressing the statistical power problem in imaging studies is nontrivial. We depicted DD as a heterogeneous disease, and the MRI findings also reported the same to date (Figure 1). Generally speaking, the estimation of the minimum sample size required to highlight structural or functional imaging alteration is prohibitive. One may argue that some areas, that have been reported more consistently in literature, are more consistently altered and thus require a smaller sample size to be detected. The problem is worsened by the variability introduced by MRI techniques and methods as the multiple comparisons correction, that greatly limits the comparability of results across studies. New candidate gene–candidate phenotype imaging association studies should integrate investigations of the effects of selective genetic variants upon neuroanatomical pathways underlying the specific reading-related cognitive and sensorial processes each gene is supposed to target by applying the most sensitive and robust neuroimaging techniques. Future hypothesis-driven imaging–genetic studies should therefore take advantage of recent genetic findings in both animal and human studies to focus their attention on innovative interdisciplinary analyses of well-defined, specific cognitive and sensorial, imaging and selective genetic data. In this way, the effect of a known genetic diversity, naturally occurring among human populations, is studied by brain imaging to determine whether one of its forms can cause a difference in the level of such cognitive/sensorial phenotypes and hence could make people more vulnerable to neurodevelopmental disorders.^4^ A fruitful outcome is particularly possible when fMRI is used to examine the neurobiological effect of a well-validated gene. If DD-candidate genetic variants are selectively associated with inter-individual variation in one of the reading-related processes at brain level, children carrying these genetic variants would be considered as ‘biologically at-risk’. Early identification of these children would be crucial to defining adequate and well-timed prevention strategies.^197, 242^ Furthermore, candidate gene–candidate phenotype might be fundamental to understanding the relationship between traditional diagnostic categories and the new classifications of mental disorders based on dimensions of observable behavior and neurobiological measures.^186, 187, 195, 196, 198^ Neuroimaging may provide evidence for or against existing theories, or provide unique and sensitive insight unexplained solely by behavioral measures.^198^ Although producing interesting results, the hypothesis-driven approach of imaging genetics represents a way for validation/replication studies of selective genes and do not reveal other genetic contributors to the overall neurobehavioral reading deficits nor the imaging phenotype changes associated with DD.^4, 12, 31^ By implementing a ‘gene hunting’ strategy,^4^ hypothesis-free approach, similar to those commonly seen in human genetics such as genome-wide association studies and new DNA sequencing technologies, could detect common variants with small effect sizes and could reveal new genes and pathways, rare and *de novo* variants, that contribute to alterations in brain imaging phenotypes, and how they contribute to the ultimate neurobehavioral phenotypes.^12, 31, 235^ However, the question that arises from imaging–genetics as a hypothesis-free field is how to use and analyze such large and diverse datasets. Data reduction or hypothesis-free processing methods, such as parallel independent component analysis,^201, 202^ multivariate pattern analysis,^227^ endophenotype ranking value,^243^ polygenic risk score,^244^ as well as new analytical methods to collapse and/or integrate a variety of data types into relevant risk models (for example, support vector machine analysis) are potentially needed.

## Conclusion

This review aimed to highlight the promising imaging–genetics approach as a way to unravel new insights behind the pathophysiology of reading (dis)ability. As the presence of putative functional genetic variants influencing the expression of some of the DD-candidate genes has been provided and as genetic associations with specific, well-defined cognitive/sensorial mechanisms have been reported, current knowledge of genetics of DD could help target imaging more selectively. The integration of particular cognitive/sensorial, selective genetic and imaging data, as well as the implementation of candidate gene–candidate phenotype imaging association studies would result in a better consideration of what constitutes a phenotype. Clearly, such an approach is essentially interdisciplinary given the multiple levels of analysis simultaneously achieved. Even if there are weaknesses despite strengths in this perspective, such hypothesis-driven approach in imaging–genetics as a field would lead to the optimization of criteria to diagnose DD and to the early identification of ‘biologically at-risk’ children. This means the definition of adequate and well-timed prevention strategies and the implementation of novel, specific and evidence-based remediation approach training specifically the reading-related cognitive/sensorial impairment. These insights will aid in the earlier detection of children with DD and aid their overall academic and remediation potential. Naturally, these developments should be considered in parallel with the advance made by the hypothesis-free approach that will aid in the identification of new mechanisms (genetic and imaging) that contribute to reading deficits in DD.

## Figures and Tables

**Table 1:** Imaging genetic studies in DD

Locus	*Location*	*Gene*	*Function*	*Reliability*	*Imaging*	*Results*
*DYX1*	15q21	*DYX1C1*	Neuronal migration, estrogen receptor transport, and cilia structure and function	Ten independent samples (Finnish, British, two Italian, German, Canadian, Australian, American, Indian, Chinese)	Structural	rs3743205 is significantly correlated with the inferior cerebellar network in both subjects with SKZ and in controls; the magnitude of the relationship did not differ between groups. On the contrary, the gender-matched subsample showed a stronger correlation in subjects with SKZ compared with controls (Jamadar *et al.*^201^) rs3743204 is significantly associated with WM volume of the temporo-parietal region containing WM pathways connecting the MTG with the inferior parietal lobe, that is, the SLF and the posterior part of CC (Darki *et al.*^203, 204^)
						
*DYX2*	6p22.3-p21.3	*DCDC2*	Neurite outgrowth, neuronal migration and ciliary functions	Ten independent samples (two American, two British, German, Australian, Canadian, Italian, Chinese, Indian)	Structural	rs793842 is significantly associated with WM volume of the temporo-parietal region containing WM pathways connecting the MTG with the inferior parietal lobe, that is, the SLF and the posterior part of CC (Darki *et al.*^203, 204^) rs793842 is significantly associated with the thickness of left AG and SG as well as the left LOC (Darki *et al.*^204^) rs1087266 is significantly correlated with the superior prefrontal, occipital and temporal networks in subjects with SKZ but not in controls. rs793862 is significantly correlated with the superior cerebellar network in both subjects with SKZ and in controls; the magnitude of the relationship did not differ between groups. On the contrary, in the gender-matched subsample the correlation in subjects with SKZ do not reach significance while it is significant in controls (Jamadar *et al.*^201^) *DCDC2*d is significantly correlated with higher GM volumes in left TG, FG, H/PHG, IOPG, IFG, and IMG (Meda *et al.*^200^) *DCDC2*d is associated with FA decreases in the bilateral ILF and in the genu of the CC in subjects with DD, and with FA reductions in the genu of the CC bilaterally and in the body of the CC in the right hemisphere, in the left ILF, AF and IFOF, and in the right IFOF, and in the body and splenium of CC in controls (Marino *et al.*^207^)
					Functional	rs1087266 and rs793862 significantly correlate with Broca-Medial-Parietal network in both subjects with SKZ and controls (Jamadar *et al.*^202^) During PC, BV677278 complex tandem repeat is associated with left AIPL and right LOTG. During AC, BV677278 complex tandem repeat is associated with right LOTG. During reading tasks, BV677278 complex tandem repeat is nominally associated with the SACG, PCG, left PCL and IFG, and rs2143340 with the bilateral AIPL (Cope *et al.*^199^)
		*KIAA0319*	Neuronal migration and ciliary functions		Structural	rs4504469 is significantly correlated with the superior cerebellar network in both subjects with SKZ and in controls; the magnitude of the relationship did not differ between groups. On the contrary, in the gender-matched subsample the correlation in subjects with SKZ do not reach significance while it is significant in controls (Jamadar *et al.*^201^) rs6935076 is significantly associated with WM volume of the temporo-parietal region containing WM pathways connecting the MTG with the inferior parietal lobe, that is, the SLF and the posterior part of CC (Darki *et al.*^203, 204^) rs9461045 is associated with cortical thickness in the left orbitofrontal region and FA in the CC (Eicher *et al.*^208^)
					Functional	rs17243157 is associated with asymmetry in functional activation of the STS (Pinel *et al.*^205^) rs2038136 and rs2038137 significantly correlate with the left Broca-superior/inferior parietal network in controls, and with the left Wernicke-fronto-occipital network in both subjects with SKZ and controls. rs4504469 is significantly correlated with the bilateral Wernicke-fronto-parietal network in controls (Jamadar *et al.*^202^)
						
*DYX3*	2p16-p15	*MRPL19 and C2ORF3*	rRNA processing	Two independent samples (Finnish and German)	Structural	rs917235 is significantly associated with WM structure in the posterior part of the CC and cingulum, connecting large parts of the cortex in the parietal, occipital and temporal lobes (Scerri *et al.*^206^) rs917235 and rs6732511 show suggestive association with cortical thickness in the left middle temporal region and cortical volume in the right fusiform region, respectively. rs2298248 is associated with cortical thickness in the right middle temporal region and with cortical volume in the right inferior temporal region (Eicher *et al.*^208^).
*DYX4*	6q11.2-q12	—	—	—	—	—
*DYX5*	3p12-q13	*ROBO1*	Axon guidance receptor regulating the connections between brain hemispheres	Four independent samples (Finnish, Australian, Italian, Indian)	—	—
*DYX6*	18p11.2	*MC5R, DYM, NEDD4L*	—	—	—	—
*DYX7*	11p15.5	—	—	—	—	—
*DYX8*	1p36-p34	*KIAA0319L*	—		—	—
*DYX9*	Xq27.2-q28	—	—	—	—	—
No locus named	12p13.1	*GRIN2B*	Neuronal pattern formation, channel function and formation of dendritic spines in hippocampal pyramidal cells	Two independent samples (German and Italian)	Functional	rs2160517, rs219931, rs11055792, rs17833967 and rs12814951 are associated with the dorsolateral prefrontal cortex activity during a working memory tasks (Pergola *et al.*^209^)
						
No locus named	7q31	*FOXP2*	Neurite growth and branching, transcriptional regulation	Two independent samples (American and German)	Functional	rs6980093 is associated with higher levels of activation in the bilateral IFG during both reading and speech listening tasks (Pinel *et al.*^205^) rs12533005 modulates the activation in occipital and inferior temporal brain areas, the AG, the insula and inferior frontal brain areas, during phonological and visual processing tasks (Wilcke *et al.*^17^)
						
No locus named	7q35	*CNTNAP2*	Neuronal connectivity at the cellular and network level, interneuron development/function, synaptic organization and activity, migration of neurons	Two independent samples (British and German)	Structural	rs7794745 is associated with altered structural brain connectivity in a general population sample (Dennis *et al.*^211^) and with reduction in GM and WM volume and FA in the cerebellum, FG, occipital and frontal cortices in subjects with ASD (Tan *et al.*^214^) rs2710126 is associated with FA in the uncinate fasciculus in subjects with SKZ (von Hohenberg *et al.*^213^)
					Functional	rs2710102 is associated with modulation of frontal lobar connectivity in subjects with ASD autism spectrum disorder (Scott Van-Zeeland *et al.*^215^), and with increased brain activation in BA7, BA44 and BA21 during a language processing task in healthy individuals (Whalley *et al.*^212^) rs7794745 is associated with brain activation in BA7, BA44 and BA21 during a language processing task in healthy individuals (Whalley *et al.*^212^)

Abbreviations: AC, auditory categorization; AF, arcuate fasciculus; AG, angular gyrus; AIPL, anterior inferior parietal lobe; ASD, autism spectrum disorder; BA, Brodman's area; CC, corpus callosum; *DCDC2*d, deletion in intron 2 of the *DCDC2* gene; DD, developmental dyslexia; FA, fractional anisotropy; FG, fusiform gyrus; GM, gray matter; H/PHG, hippocampal/parahippocampal gyrus; IFG, inferior frontal gyrus; IFOF, inferior fronto-occipital fasciculus; ILF, inferior longitudinal fasciculus; IMG, inferior medial gyrus; IOPG, inferior occipito-parietal gyrus; LOC, lateral occipital cortex; LOTG, lateral occipital temporal gyrus; MTG, middle temporal gyrus; PC, print categorization; PCG, posterior cingulate gyrus; PCL, paracentral lobule; SACG, superior anterior cingulate gyrus; SG, supramarginal gyrus; SKZ, schizophrenia; SLF, superior longitudinal fasciculus; STS, superior temporal sulcus; TG, temporal gyrus; WM, white matter.

## References

[bib1] Norton ES, Wolf M. Rapid automatized naming (RAN) and reading fluency: implications for understanding and treatment of reading disabilities. Annu Rev Psychol 2012; 63: 427–452. 21838545 10.1146/annurev-psych-120710-100431

[bib2] Peterson RL, Pennington BF. Developmental dyslexia. Annu Rev Clin Psychol 2015; 11: 283–307. 25594880 10.1146/annurev-clinpsy-032814-112842

[bib3] American Psychiatric AssociationDiagnostic and Statistical Manual of Mental Disorders, 5th edn. Washington, DC, 2013.

[bib4] Arslan A. Genes, brains, and behavior: imaging genetics for neuropsychiatric disorders. J Neuropsychiatry Clin Neurosci 2015; 27: 81–92. 25751509 10.1176/appi.neuropsych.13080185

[bib5] Hallgren B. Specific dyslexia (congenital word-blindness); a clinical and genetic study. Acta Psychiatr Neurol 1950; 65: 1–287. 14846691

[bib6] Fisher SE, DeFries JC. Developmental dyslexia: genetic dissection of a complex cognitive trait. Nat Rev 2002; 3: 767–780. 10.1038/nrn936 12360321

[bib7] Plomin R, Kovas Y. Generalist genes and learning disabilities. Psychol Bull 2005; 131: 592–617. 16060804 10.1037/0033-2909.131.4.592

[bib8] Scerri TS, Schulte-Korne G. Genetics of developmental dyslexia. Eur Child Adolesc Psychiatry 2010; 19: 179–197. 20091194 10.1007/s00787-009-0081-0

[bib9] Carrion-Castillo A, Franke B, Fisher SE. Molecular genetics of dyslexia: an overview. Dyslexia 2013; 19: 214–240. 24133036 10.1002/dys.1464

[bib10] Zhang Y, Li J, Song S, Tardif T, Burmeister M, Villafuerte SM et al. Association of DCDC2 polymorphisms with normal variations in reading abilities in a Chinese population. PLoS One 2016; 11: e0153603. 27100778 10.1371/journal.pone.0153603 PMC4839751

[bib11] Zhao H, Chen Y, Zhang B-P, Zuo P-X. KIAA0319 gene polymorphisms are associated with developmental dyslexia in Chinese Uyghur children. J Hum Genet 2016; 61: 745–752. 27098879 10.1038/jhg.2016.40 PMC4999827

[bib12] Eicher JD, Gruen JR. Imaging-genetics in dyslexia: connecting risk genetic variants to brain neuroimaging and ultimately to reading impairments. Mol Genet Metab 2013; 110: 201–212. 23916419 10.1016/j.ymgme.2013.07.001 PMC3800223

[bib13] Skeide MA, Kraft I, Müller B, Schaadt G, Neef NE, Brauer J et al. NRSN1 associated grey matter volume of the visual word form area reveals dyslexia before school. Brain 2016; 139: 2792–2803. 27343255 10.1093/brain/aww153

[bib14] Pagnamenta AT, Bacchelli E, de Jonge MV, Mirza G, Scerri TS, Minopoli F et al. Characterization of a family with rare deletions in CNTNAP5 and DOCK4 suggests novel risk loci for autism and dyslexia. Biol Psychiatry 2010; 68: 320–328. 20346443 10.1016/j.biopsych.2010.02.002 PMC2941017

[bib15] Newbury DF, Paracchini S, Scerri TS, Winchester L, Addis L, Richardson AJ et al. Investigation of dyslexia and SLI risk variants in reading- and language-impaired subjects. Behav Genet 2011; 41: 90–104. 21165691 10.1007/s10519-010-9424-3 PMC3029677

[bib16] Peter B, Raskind WH, Matsushita M, Lisowski M, Vu T, Berninger VW et al. Replication of CNTNAP2 association with nonword repetition and support for FOXP2 association with timed reading and motor activities in a dyslexia family sample. J Neurodev Disord 2011; 3: 39–49. 21484596 10.1007/s11689-010-9065-0 PMC3163991

[bib17] Wilcke A, Ligges C, Burkhardt J, Alexander M, Wolf C, Quente E et al. Imaging genetics of FOXP2 in dyslexia. Eur J Hum Genet 2012; 20: 224–229. 21897444 10.1038/ejhg.2011.160 PMC3260915

[bib18] Ludwig KU, Roeske D, Herms S, Schumacher J, Warnke A, Plume E et al. Variation in GRIN2B contributes to weak performance in verbal short-term memory in children with dyslexia. Am J Med Genet B Neuropsychiatr Genet Off Publ Int Soc Psychiatr Genet 2010; 153B: 503–511. 10.1002/ajmg.b.31007 19591125

[bib19] Konig IR, Schumacher J, Hoffmann P, Kleensang A, Ludwig KU, Grimm T et al. Mapping for dyslexia and related cognitive trait loci provides strong evidence for further risk genes on chromosome 6p21. Am J Med Genet B, Neuropsychiatr Genet 2011; 156B: 36–43. 21184582 10.1002/ajmg.b.31135

[bib20] Mascheretti S, Facoetti A, Giorda R, Beri S, Riva V, Trezzi V et al. GRIN2B mediates susceptibility to intelligence quotient and cognitive impairments in developmental dyslexia. Psychiatr Genet 2015; 25: 9–20. 25426763 10.1097/YPG.0000000000000068

[bib21] Scerri TS, Morris AP, Buckingham LL, Newbury DF, Miller LL, Monaco AP et al. DCDC2, KIAA0319 and CMIP are associated with reading-related traits. Biol Psychiatry 2011; 70: 237–245. 21457949 10.1016/j.biopsych.2011.02.005 PMC3139836

[bib22] Matsson H, Huss M, Persson H, Einarsdottir E, Tiraboschi E, Nopola-Hemmi J et al. Polymorphisms in DCDC2 and S100B associate with developmental dyslexia. J Hum Genet 2015; 60: 399–401. 25877001 10.1038/jhg.2015.37 PMC4521290

[bib23] Kong R, Shao S, Wang J, Zhang X, Guo S, Zou L et al. Genetic variant in DIP2A gene is associated with developmental dyslexia in Chinese population. Am J Med Genet B Neuropsychiatr Genet 2016; 171B: 203–208. 26452339 10.1002/ajmg.b.32392

[bib24] Veerappa AM, Saldanha M, Padakannaya P, Ramachandra NB. Family-based genome-wide copy number scan identifies five new genes of dyslexia involved in dendritic spinal plasticity. J Hum Genet 2013; 58: 539–547. 23677055 10.1038/jhg.2013.47

[bib25] Massinen S, Wang J, Laivuori K, Bieder A, Tapia Paez I, Jiao H et al. Genomic sequencing of a dyslexia susceptibility haplotype encompassing ROBO1. J Neurodev Disord 2016; 8: 4. 26877820 10.1186/s11689-016-9136-y PMC4751651

[bib26] Roeske D, Ludwig KU, Neuhoff N, Becker J, Bartling J, Bruder J et al. First genome-wide association scan on neurophysiological endophenotypes points to trans-regulation effects on SLC2A3 in dyslexic children. Mol Psychiatry 2011; 16: 97–107. 19786962 10.1038/mp.2009.102

[bib27] Massinen S, Hokkanen ME, Matsson H, Tammimies K, Tapia-Paez I, Dahlstrom-Heuser V et al. Increased expression of the dyslexia candidate gene DCDC2 affects length and signaling of primary cilia in neurons. PLoS One 2011; 6: e20580. 21698230 10.1371/journal.pone.0020580 PMC3116825

[bib28] Luciano M, Evans DM, Hansell NK, Medland SE, Montgomery GW, Martin NG et al. A genome-wide association study for reading and language abilities in two population cohorts. Genes Brain Behav 2013; 12: 645–652. 23738518 10.1111/gbb.12053 PMC3908370

[bib29] Gialluisi A, Newbury DF, Wilcutt EG, Olson RK, DeFries JC, Brandler WM et al. Genome-wide screening for DNA variants associated with reading and language traits. Genes Brain Behav 2014; 13: 686–701. 25065397 10.1111/gbb.12158 PMC4165772

[bib30] Einarsdottir E, Svensson I, Darki F, Peyrard-Janvid M, Lindvall JM, Ameur A et al. Mutation in CEP63 co-segregating with developmental dyslexia in a Swedish family. Hum Genet 2015; 134: 1239–1248. 26400686 10.1007/s00439-015-1602-1 PMC4628622

[bib31] Graham SA, Fisher SE. Decoding the genetics of speech and language. Curr Opin Neurobiol 2013; 23: 43–51. 23228431 10.1016/j.conb.2012.11.006

[bib32] Taipale M, Kaminen N, Nopola-Hemmi J, Haltia T, Myllyluoma B, Lyytinen H et al. A candidate gene for developmental dyslexia encodes a nuclear tetratricopeptide repeat domain protein dynamically regulated in brain. Proc Natl Acad Sci USA 2003; 100: 11553–11558. 12954984 10.1073/pnas.1833911100 PMC208796

[bib33] Marino C, Citterio A, Giorda R, Facoetti A, Menozzi G, Vanzin L et al. Association of short-term memory with a variant within DYX1C1 in developmental dyslexia. Genes Brain Behav 2007; 6: 640–646. 17309662 10.1111/j.1601-183X.2006.00291.x

[bib34] Marino C, Meng H, Mascheretti S, Rusconi M, Cope N, Giorda R et al. DCDC2 genetic variants and susceptibility to developmental dyslexia. Psychiatr Genet 2012; 22: 25–30. 21881542 10.1097/YPG.0b013e32834acdb2 PMC3232293

[bib35] Wigg KG, Couto JM, Feng Y, Anderson B, Cate-Carter TD, Macciardi F et al. Support for EKN1 as the susceptibility locus for dyslexia on 15q21. Mol Psychiatry 2004; 9: 1111–1121. 15249932 10.1038/sj.mp.4001543

[bib36] Scerri TS, Fisher SE, Francks C, MacPhie IL, Paracchini S, Richardson AJ et al. Putative functional alleles of DYX1C1 are not associated with dyslexia susceptibility in a large sample of sibling pairs from the UK. J Med Genet 2004; 41: 853–857. 15520411 10.1136/jmg.2004.018341 PMC1735619

[bib37] Brkanac Z, Chapman NH, Matsushita MM, Chun L, Nielsen K, Cochrane E et al. Evaluation of candidate genes for DYX1 and DYX2 in families with dyslexia. Am J Med Genet B, Neuropsychiatr Genet 2007; 144B: 556–560. 17450541 10.1002/ajmg.b.30471

[bib38] Dahdouh F, Anthoni H, Tapia-Paez I, Peyrard-Janvid M, Schulte-Korne G, Warnke A et al. Further evidence for DYX1C1 as a susceptibility factor for dyslexia. Psychiatr Genet 2009; 19: 59–63. 19240663 10.1097/YPG.0b013e32832080e1

[bib39] Bates TC, Lind PA, Luciano M, Montgomery GW, Martin NG, Wright MJ. Dyslexia and DYX1C1: deficits in reading and spelling associated with a missense mutation. Mol Psychiatry 2010; 15: 1190–1196. 19901951 10.1038/mp.2009.120

[bib40] Lim CK, Ho CS, Chou CH, Waye MM. Association of the rs3743205 variant of DYX1C1 with dyslexia in Chinese children. Behav Brain Funct 2011; 7: 16. 21599957 10.1186/1744-9081-7-16 PMC3123182

[bib41] Paracchini S, Ang QW, Stanley FJ, Monaco AP, Pennell CE, Whitehouse AJ. Analysis of dyslexia candidate genes in the Raine cohort representing the general Australian population. Genes Brain Behav 2011; 10: 158–165. 20846247 10.1111/j.1601-183X.2010.00651.x PMC3084500

[bib42] Tran C, Gagnon F, Wigg KG, Feng Y, Gomez L, Cate-Carter TD et al. A family-based association analysis and meta-analysis of the reading disabilities candidate gene DYX1C1. Am J Med Genet B, Neuropsychiatr Genet 2013; 162B: 146–156. 23341075 10.1002/ajmg.b.32123 PMC5381964

[bib43] Bellini G, Bravaccio C, Calamoneri F, Cocuzza MD, Fiorillo P, Gagliano A et al. No evidence for association between dyslexia and DYX1C1 functional variants in a group of children and adolescents from Southern Italy. J Mol Neurosci 2005; 27: 311–314. 16280601 10.1385/jmn:27:3:311

[bib44] Francks C, Paracchini S, Smith SD, Richardson AJ, Scerri TS, Cardon LR et al. A 77-kilobase region of chromosome 6p22.2 is associated with dyslexia in families from the United Kingdom and from the United States. Am J Hum Genet 2004; 75: 1046–1058. 15514892 10.1086/426404 PMC1182140

[bib45] Cope N, Harold D, Hill G, Moskvina V, Stevenson J, Holmans P et al. Strong evidence that KIAA0319 on chromosome 6p is a susceptibility gene for developmental dyslexia. Am J Hum Genet 2005; 76: 581–591. 15717286 10.1086/429131 PMC1199296

[bib46] Harold D, Paracchini S, Scerri T, Dennis M, Cope N, Hill G et al. Further evidence that the KIAA0319 gene confers susceptibility to developmental dyslexia. Mol Psychiatry 2006; 11: 1085–1091. 17033633 10.1038/sj.mp.4001904

[bib47] Dennis MY, Paracchini S, Scerri TS, Prokunina-Olsson L, Knight JC, Wade-Martins R et al. A common variant associated with dyslexia reduces expression of the KIAA0319 gene. PLoS Genet 2009; 5: e1000436. 19325871 10.1371/journal.pgen.1000436 PMC2653637

[bib48] Luciano M, Lind PA, Duffy DL, Castles A, Wright MJ, Montgomery GW et al. A haplotype spanning KIAA0319 and TTRAP is associated with normal variation in reading and spelling ability. Biol Psychiatry 2007; 62: 811–817. 17597587 10.1016/j.biopsych.2007.03.007

[bib49] Paracchini S, Steer CD, Buckingham LL, Morris AP, Ring S, Scerri T et al. Association of the KIAA0319 dyslexia susceptibility gene with reading skills in the general population. Am J Psychiatry 2008; 165: 1576–1584. 18829873 10.1176/appi.ajp.2008.07121872

[bib50] Meng H, Smith SD, Hager K, Held M, Liu J, Olson RK et al. DCDC2 is associated with reading disability and modulates neuronal development in the brain. Proc Natl Acad Sci USA 2005; 102: 17053–17058. 16278297 10.1073/pnas.0508591102 PMC1278934

[bib51] Meng H, Powers NR, Tang L, Cope NA, Zhang PX, Fuleihan R et al. A dyslexia-associated variant in DCDC2 changes gene expression. Behav Genet 2011; 41: 58–66. 21042874 10.1007/s10519-010-9408-3 PMC3053575

[bib52] Powers NR, Eicher JD, Butter F, Kong Y, Miller LL, Ring SM et al. Alleles of a polymorphic ETV6 binding site in DCDC2 confer risk of reading and language impairment. Am J Hum Genet 2013; 93: 19–28. 23746548 10.1016/j.ajhg.2013.05.008 PMC3710765

[bib53] Ludwig KU, Schumacher J, Schulte-Korne G, Konig IR, Warnke A, Plume E et al. Investigation of the DCDC2 intron 2 deletion/compound short tandem repeat polymorphism in a large German dyslexia sample. Psychiatr Genet 2008; 18: 310–312. 19018237 10.1097/YPG.0b013e3283063a78 PMC9748830

[bib54] Wilcke A, Weissfuss J, Kirsten H, Wolfram G, Boltze J, Ahnert P. The role of gene DCDC2 in German dyslexics. Ann Dyslexia 2009; 59: 1–11. 19238550 10.1007/s11881-008-0020-7

[bib55] Lind PA, Luciano M, Wright MJ, Montgomery GW, Martin NG, Bates TC. Dyslexia and DCDC2: normal variation in reading and spelling is associated with DCDC2 polymorphisms in an Australian population sample. Eur J Hum Genet 2010; 18: 668–673. 20068590 10.1038/ejhg.2009.237 PMC2987340

[bib56] Flint J, Timpson N, Munafo M. Assessing the utility of intermediate phenotypes for genetic mapping of psychiatric disease. Trends Neurosci 2014; 37: 733–741. 25216981 10.1016/j.tins.2014.08.007 PMC4961231

[bib57] Cicchini GM, Marino C, Mascheretti S, Perani D, Morrone MC. Strong motion deficits in dyslexia associated with DCDC2 gene alteration. J Neurosci 2015; 35: 8059–8064. 26019324 10.1523/JNEUROSCI.5077-14.2015 PMC4888943

[bib58] Gori S, Mascheretti S, Giora E, Ronconi L, Ruffino M, Quadrelli E et al. The DCDC2 intron 2 deletion impairs illusory motion perception unveiling the selective role of magnocellular-dorsal stream in reading (dis)ability. Cereb Cortex 2015; 25: 1685–1695. 25270309 10.1093/cercor/bhu234

[bib59] Strauss KA, Puffenberger EG, Huentelman MJ, Gottlieb S, Dobrin SE, Parod JM et al. Recessive symptomatic focal epilepsy and mutant contactin-associated protein-like 2. N Engl J Med 2006; 354: 1370–1377. 16571880 10.1056/NEJMoa052773

[bib60] Jackman C, Horn ND, Molleston JP, Sokol DK. Gene associated with seizures, autism, and hepatomegaly in an Amish girl. Pediatr Neurol 2009; 40: 310–313. 19302947 10.1016/j.pediatrneurol.2008.10.013

[bib61] Whitford KL, Marillat V, Stein E, Goodman CS, Tessier-Lavigne M, Chédotal A et al. Regulation of cortical dendrite development by Slit-Robo interactions. Neuron 2002; 33: 47–61. 11779479 10.1016/s0896-6273(01)00566-9

[bib62] Hannula-Jouppi K, Kaminen-Ahola N, Taipale M, Eklund R, Nopola-Hemmi J, Kaariainen H et al. The axon guidance receptor gene ROBO1 is a candidate gene for developmental dyslexia. PLoS Genet 2005; 1: e50. 16254601 10.1371/journal.pgen.0010050 PMC1270007

[bib63] Andrews W, Liapi A, Plachez C, Camurri L, Zhang J, Mori S et al. Robo1 regulates the development of major axon tracts and interneuron migration in the forebrain. Development 2006; 133: 2243–2252. 16690755 10.1242/dev.02379

[bib64] Wang Y, Paramasivam M, Thomas A, Bai J, Kaminen-Ahola N, Kere J et al. DYX1C1 functions in neuronal migration in developing neocortex. Neuroscience 2006; 143: 515–522. 16989952 10.1016/j.neuroscience.2006.08.022

[bib65] Rosen GD, Bai J, Wang Y, Fiondella CG, Threlkeld SW, LoTurco JJ et al. Disruption of neuronal migration by RNAi of Dyx1c1 results in neocortical and hippocampal malformations. Cereb Cortex 2007; 17: 2562–2572. 17218481 10.1093/cercor/bhl162 PMC3742088

[bib66] Threlkeld SW, McClure MM, Bai J, Wang Y, LoTurco JJ, Rosen GD et al. Developmental disruptions and behavioral impairments in rats following *in utero* RNAi of Dyx1c1. Brain Res Bull 2007; 71: 508–514. 17259020 10.1016/j.brainresbull.2006.11.005 PMC1893003

[bib67] Burbridge TJ, Wang Y, Volz AJ, Peschansky VJ, Lisann L, Galaburda AM et al. Postnatal analysis of the effect of embryonic knockdown and overexpression of candidate dyslexia susceptibility gene homolog Dcdc2 in the rat. Neuroscience 2008; 152: 723–733. 18313856 10.1016/j.neuroscience.2008.01.020 PMC2424111

[bib68] Akashi K, Kakizaki T, Kamiya H, Fukaya M, Yamasaki M, Abe M et al. NMDA receptor GluN2B (GluR epsilon 2/NR2B) subunit is crucial for channel function, postsynaptic macromolecular organization, and actin cytoskeleton at hippocampal CA3 synapses. J Neurosci 2009; 29: 10869–10882. 19726645 10.1523/JNEUROSCI.5531-08.2009 PMC6665524

[bib69] Velayos-Baeza A, Levecque C, Kobayashi K, Holloway ZG, Monaco AP. The dyslexia-associated KIAA0319 protein undergoes proteolytic processing with {gamma}-secretase-independent intramembrane cleavage. J Biol Chem 2010; 285: 40148–40162. 20943657 10.1074/jbc.M110.145961 PMC3000997

[bib70] Peschansky VJ, Burbridge TJ, Volz AJ, Fiondella C, Wissner-Gross Z, Galaburda AM et al. The effect of variation in expression of the candidate dyslexia susceptibility gene homolog Kiaa0319 on neuronal migration and dendritic morphology in the rat. Cereb Cortex 2010; 20: 884–897. 19679544 10.1093/cercor/bhp154 PMC2837091

[bib71] Currier TA, Etchegaray MA, Haight JL, Galaburda AM, Rosen GD. The effects of embryonic knockdown of the candidate dyslexia susceptibility gene homologue Dyx1c1 on the distribution of GABAergic neurons in the cerebral cortex. Neuroscience 2011; 172: 535–546. 21070838 10.1016/j.neuroscience.2010.11.002 PMC3010415

[bib72] Poon MW, Tsang WH, Chan SO, Li HM, Ng HK, Waye MM. Dyslexia-associated kiaa0319-like protein interacts with axon guidance receptor nogo receptor 1. Cell Mol Neurobiol 2011; 31: 27–35. 20697954 10.1007/s10571-010-9549-1 PMC11498553

[bib73] Vernes SC, Oliver PL, Spiteri E, Lockstone HE, Puliyadi R, Taylor JM et al. Foxp2 regulates gene networks implicated in neurite outgrowth in the developing brain. PLoS Genet 2011; 7: e1002145. 21765815 10.1371/journal.pgen.1002145 PMC3131290

[bib74] Szalkowski CE, Hinman JR, Threlkeld SW, Wang Y, LePack A, Rosen GD et al. Persistent spatial working memory deficits in rats following *in utero* RNAi of Dyx1c1. Genes Brain Behav 2011; 10: 244–252. 20977651 10.1111/j.1601-183X.2010.00662.x PMC3041839

[bib75] Szalkowski CE, Fiondella CG, Galaburda AM, Rosen GD, Loturco JJ, Fitch RH. Neocortical disruption and behavioral impairments in rats following *in utero* RNAi of candidate dyslexia risk gene Kiaa0319. Int J Dev Neurosci 2012; 30: 293–302. 22326444 10.1016/j.ijdevneu.2012.01.009 PMC3516384

[bib76] Szalkowski CE, Booker AB, Truong DT, Threlkeld SW, Rosen GD, Fitch RH. Knockdown of the candidate dyslexia susceptibility gene homolog dyx1c1 in rodents: effects on auditory processing, visual attention, and cortical and thalamic anatomy. Dev Neurosci 2013; 35: 50–68. 23594585 10.1159/000348431 PMC3980864

[bib77] Tsui D, Vessey JP, Tomita H, Kaplan DR, Miller FD. FoxP2 regulates neurogenesis during embryonic cortical development. J Neurosci 2013; 33: 244–258. 23283338 10.1523/JNEUROSCI.1665-12.2013 PMC6618635

[bib78] Tarkar A, Loges NT, Slagle CE, Francis R, Dougherty GW, Tamayo J V et al. DYX1C1 is required for axonemal dynein assembly and ciliary motility. Nat Genet 2013; 45: 995–1003. 23872636 10.1038/ng.2707 PMC4000444

[bib79] Kato M, Okanoya K, Koike T, Sasaki E, Okano H, Watanabe S et al. Human speech- and reading-related genes display partially overlapping expression patterns in the marmoset brain. Brain Lang 2014; 133: 26–38. 24769279 10.1016/j.bandl.2014.03.007

[bib80] Martinez-Garay I, Guidi LG, Holloway ZG, Bailey MAG, Lyngholm D, Schneider T et al. Normal radial migration and lamination are maintained in dyslexia-susceptibility candidate gene homolog Kiaa0319 knockout mice. Brain Struct Funct 2016; doi:10.1007/s00429-016-1282-1. 10.1007/s00429-016-1282-1 PMC5368214 27510895

[bib81] Tammimies K, Bieder A, Lauter G, Sugiaman-Trapman D, Torchet R, Hokkanen M-E et al. Ciliary dyslexia candidate genes DYX1C1 and DCDC2 are regulated by Regulatory Factor (RF) X transcription factors through X-box promoter motifs. FASEB J 2016; 30: 3578–3587. 27451412 10.1096/fj.201500124RR PMC5024701

[bib82] Peñagarikano O, Abrahams BS, Herman EI, Winden KD, Gdalyahu A, Dong H et al. Absence of CNTNAP2 leads to epilepsy, neuronal migration abnormalities, and core autism-related deficits. Cell 2011; 147: 235–246. 21962519 10.1016/j.cell.2011.08.040 PMC3390029

[bib83] Rendall AR, Tarkar A, Contreras-Mora HM, LoTurco JJ, Fitch RH. Deficits in learning and memory in mice with a mutation of the candidate dyslexia susceptibility gene Dyx1c1. Brain Lang 2015; doi:10.1016/j.bandl.2015.04.008. 10.1016/j.bandl.2015.04.008 PMC4646737 25989970

[bib84] Szalkowski CE, Fiondella CF, Truong DT, Rosen GD, LoTurco JJ, Fitch RH. The effects of Kiaa0319 knockdown on cortical and subcortical anatomy in male rats. Int J Dev Neurosci 2013; 31: 116–122. 23220223 10.1016/j.ijdevneu.2012.11.008 PMC3689304

[bib85] Centanni TM, Booker AB, Sloan AM, Chen F, Maher BJ, Carraway RS et al. Knockdown of the dyslexia-associated gene Kiaa0319 impairs temporal responses to speech stimuli in rat primary auditory cortex. Cereb Cortex 2014; 24: 1753–1766. 23395846 10.1093/cercor/bht028 PMC4051891

[bib86] Wang Y, Yin X, Rosen G, Gabel L, Guadiana SM, Sarkisian MR et al. Dcdc2 knockout mice display exacerbated developmental disruptions following knockdown of doublecortin. Neuroscience 2011; 190: 398–408. 21689730 10.1016/j.neuroscience.2011.06.010 PMC3170724

[bib87] Truong DT, Che A, Rendall AR, Szalkowski CE, LoTurco JJ, Galaburda AM et al. Mutation of Dcdc2 in mice leads to impairments in auditory processing and memory ability. Genes Brain Behav 2014; 13: 802–811. 25130614 10.1111/gbb.12170 PMC4241168

[bib88] Centanni TM, Booker AB, Chen F, Sloan AM, Carraway RS, Rennaker RL et al. Knockdown of dyslexia-gene Dcdc2 interferes with speech sound discrimination in continuous streams. J Neurosci 2016; 36: 4895–4906. 27122044 10.1523/JNEUROSCI.4202-15.2016 PMC4846679

[bib89] Schito AM, Pizzuti A, Di Maria E, Schenone A, Ratti A, Defferrari R et al. mRNA distribution in adult human brain of GRIN2B, a N-methyl-D-aspartate (NMDA) receptor subunit. Neurosci Lett 1997; 239: 49–53. 9547169 10.1016/s0304-3940(97)00853-7

[bib90] Cull-Candy S, Brickley S, Farrant M. NMDA receptor subunits: diversity, development and disease. Curr Opin Neurobiol 2001; 11: 327–335. 11399431 10.1016/s0959-4388(00)00215-4

[bib91] Kim MJ, Dunah AW, Wang YT, Sheng M. Differential roles of NR2A- and NR2B-containing NMDA receptors in Ras-ERK signaling and AMPA receptor trafficking. Neuron 2005; 46: 745–760. 15924861 10.1016/j.neuron.2005.04.031

[bib92] Che A, Girgenti MJ, LoTurco J. The dyslexia-associated gene DCDC2 is required for spike-timing precision in mouse neocortex. Biol Psychiatry 2014; 76: 387–396. 24094509 10.1016/j.biopsych.2013.08.018 PMC4025976

[bib93] Che A, Truong DT, Fitch RH, LoTurco JJ. Mutation of the dyslexia-associated gene Dcdc2 enhances glutamatergic synaptic transmission between layer 4 neurons in mouse neocortex. Cereb Cortex 2015; 26: 3705–3718. 26250775 10.1093/cercor/bhv168 PMC5004750

[bib94] Lai CS, Fisher SE, Hurst JA, Vargha-Khadem F, Monaco AP. A forkhead-domain gene is mutated in a severe speech and language disorder. Nature 2001; 413: 519–523. 11586359 10.1038/35097076

[bib95] Fisher SE, Vargha-Khadem F, Watkins KE, Monaco AP, Pembrey ME. Localisation of a gene implicated in a severe speech and language disorder. Nat Genet 1998; 18: 168–170. 9462748 10.1038/ng0298-168

[bib96] Shu W, Cho JY, Jiang Y, Zhang M, Weisz D, Elder GA et al. Altered ultrasonic vocalization in mice with a disruption in the Foxp2 gene. Proc Natl Acad Sci USA 2005; 102: 9643–9648. 15983371 10.1073/pnas.0503739102 PMC1160518

[bib97] Fujita E, Tanabe Y, Shiota A, Ueda M, Suwa K, Momoi MY et al. Ultrasonic vocalization impairment of Foxp2 (R552H) knockin mice related to speech-language disorder and abnormality of Purkinje cells. Proc Natl Acad Sci USA 2008; 105: 3117–3122. 18287060 10.1073/pnas.0712298105 PMC2268594

[bib98] Groszer M, Keays DA, Deacon RMJ, de Bono JP, Prasad-Mulcare S, Gaub S et al. Impaired synaptic plasticity and motor learning in mice with a point mutation implicated in human speech deficits. Curr Biol 2008; 18: 354–362. 18328704 10.1016/j.cub.2008.01.060 PMC2917768

[bib99] Kurt S, Groszer M, Fisher SE, Ehret G. Modified sound-evoked brainstem potentials in Foxp2 mutant mice. Brain Res 2009; 1289: 30–36. 19596273 10.1016/j.brainres.2009.06.092

[bib100] Kurt S, Fisher SE, Ehret G. Foxp2 mutations impair auditory-motor association learning. PLoS One 2012; 7: e33130. 22412993 10.1371/journal.pone.0033130 PMC3296769

[bib101] Gaub S, Groszer M, Fisher SE, Ehret G. The structure of innate vocalizations in Foxp2-deficient mouse pups. Genes Brain Behav 2010; 9: 390–401. 20132318 10.1111/j.1601-183X.2010.00570.x PMC2895353

[bib102] Lai CS, Gerrelli D, Monaco AP, Fisher SE, Copp AJ. FOXP2 expression during brain development coincides with adult sites of pathology in a severe speech and language disorder. Brain 2003; 126: 2455–2462. 12876151 10.1093/brain/awg247

[bib103] Vernes SC, Newbury DF, Abrahams BS, Winchester L, Nicod J, Groszer M et al. A functional genetic link between distinct developmental language disorders. N Engl J Med 2008; 359: 2337–2345. 18987363 10.1056/NEJMoa0802828 PMC2756409

[bib104] Rodenas-Cuadrado P, Ho J, Vernes SC. Shining a light on CNTNAP2: complex functions to complex disorders. Eur J Hum Genet 2014; 22: 171–178. 23714751 10.1038/ejhg.2013.100 PMC3895625

[bib105] Truong DT, Rendall AR, Castelluccio BC, Eigsti I-M, Fitch RH. Auditory processing and morphological anomalies in medial geniculate nucleus of Cntnap2 mutant mice. Behav Neurosci 2015; 129: 731–743. 26501174 10.1037/bne0000096

[bib106] Rendall AR, Truong DT, Fitch RH. Learning delays in a mouse model of autism spectrum disorder. Behav Brain Res 2016; 303: 201–207. 26873041 10.1016/j.bbr.2016.02.006 PMC4896155

[bib107] Yoshimoto R, Okawa K, Yoshida M, Ohno M, Kataoka N. Identification of a novel component C2ORF3 in the lariat-intron complex: lack of C2ORF3 interferes with pre-mRNA splicing via intron turnover pathway. Genes Cells 2014; 19: 78–87. 24304693 10.1111/gtc.12114

[bib108] Anthoni H, Zucchelli M, Matsson H, Müller-Myhsok B, Fransson I, Schumacher J et al. A locus on 2p12 containing the co-regulated MRPL19 and C2ORF3 genes is associated to dyslexia. Hum Mol Genet 2007; 16: 667–677. 17309879 10.1093/hmg/ddm009

[bib109] Kere J. The molecular genetics and neurobiology of developmental dyslexia as model of a complex phenotype. Biochem Biophys Res Commun 2014; 452: 236–243. 25078623 10.1016/j.bbrc.2014.07.102

[bib110] Galaburda AM, Sherman GF, Rosen GD, Aboitiz F, Geschwind N. Developmental dyslexia: four consecutive patients with cortical anomalies. Ann Neurol 1985; 18: 222–233. 4037763 10.1002/ana.410180210

[bib111] Galaburda AM, Kemper TL. Cytoarchitectonic abnormalities in developmental dyslexia: a case study. Ann Neurol 1979; 6: 94–100. 496415 10.1002/ana.410060203

[bib112] Vinckenbosch E, Robichon F, Eliez S. Gray matter alteration in dyslexia: converging evidence from volumetric and voxel-by-voxel MRI analyses. Neuropsychologia 2005; 43: 324–331. 15707610 10.1016/j.neuropsychologia.2004.06.023

[bib113] Silani G, Frith U, Demonet J-F, Fazio F, Perani D, Price C et al. Brain abnormalities underlying altered activation in dyslexia: a voxel based morphometry study. Brain 2005; 128: 2453–2461. 15975942 10.1093/brain/awh579

[bib114] Hoeft F, Ueno T, Reiss AL, Meyler A, Whitfield-Gabrieli S, Glover GH et al. Prediction of children’s reading skills using behavioral, functional, and structural neuroimaging measures. Behav Neurosci 2007; 121: 602–613. 17592952 10.1037/0735-7044.121.3.602

[bib115] Pernet CR, Poline JB, Demonet JF, Rousselet GA. Brain classification reveals the right cerebellum as the best biomarker of dyslexia. BMC Neurosci 2009; 10: 67. 19555471 10.1186/1471-2202-10-67 PMC2713247

[bib116] Dole M, Meunier F, Hoen M. Gray and white matter distribution in dyslexia: a VBM study of superior temporal gyrus asymmetry. PLoS One 2013; 8: e76823. 24098565 10.1371/journal.pone.0076823 PMC3788100

[bib117] Krafnick AJ, Flowers DL, Luetje MM, Napoliello EM, Eden GF. An investigation into the origin of anatomical differences in dyslexia. J Neurosci 2014; 34: 901–908. 24431448 10.1523/JNEUROSCI.2092-13.2013 PMC3891966

[bib118] Tamboer P, Scholte HS, Vorst HCM. Dyslexia and voxel-based morphometry: correlations between five behavioural measures of dyslexia and gray and white matter volumes. Ann Dyslexia 2015; 65: 121–141. 25908528 10.1007/s11881-015-0102-2 PMC4565889

[bib119] Xia Z, Hoeft F, Zhang L, Shu H. Neuroanatomical anomalies of dyslexia: disambiguating the effects of disorder, performance, and maturation. Neuropsychologia 2016; 81: 68–78. 26679527 10.1016/j.neuropsychologia.2015.12.003 PMC4790432

[bib120] Brown WE, Eliez S, Menon V, Rumsey JM, White CD, Reiss AL. Preliminary evidence of widespread morphological variations of the brain in dyslexia. Neurology 2001; 56: 781–783. 11274316 10.1212/wnl.56.6.781

[bib121] Brambati SM, Termine C, Ruffino M, Stella G, Fazio F, Cappa SF et al. Regional reductions of gray matter volume in familial dyslexia. Neurology 2004; 63: 742–745. 15326259 10.1212/01.wnl.0000134673.95020.ee

[bib122] Eckert MA, Leonard CM, Wilke M, Eckert M, Richards T, Richards A et al. Anatomical signatures of dyslexia in children: unique information from manual and voxel based morphometry brain measures. Cortex 2005; 41: 304–315. 15871596 10.1016/s0010-9452(08)70268-5

[bib123] Kronbichler M, Wimmer H, Staffen W, Hutzler F, Mair A, Ladurner G. Developmental dyslexia: gray matter abnormalities in the occipitotemporal cortex. Hum Brain Mapp 2008; 29: 613–625. 17636558 10.1002/hbm.20425 PMC6871168

[bib124] Steinbrink C, Vogt K, Kastrup A, Müller H-P, Juengling FD, Kassubek J et al. The contribution of white and gray matter differences to developmental dyslexia: insights from DTI and VBM at 3.0 T. Neuropsychologia 2008; 46: 3170–3178. 18692514 10.1016/j.neuropsychologia.2008.07.015

[bib125] Liu L, You W, Wang W, Guo X, Peng D, Booth J. Altered brain structure in Chinese dyslexic children. Neuropsychologia 2013; 51: 1169–1176. 23542499 10.1016/j.neuropsychologia.2013.03.010

[bib126] Stoodley CJ. Distinct regions of the cerebellum show gray matter decreases in autism, ADHD, and developmental dyslexia. Front Syst Neurosci 2014; 8: 92. 24904314 10.3389/fnsys.2014.00092 PMC4033133

[bib127] Klingberg T, Hedehus M, Temple E, Salz T, Gabrieli JD, Moseley ME et al. Microstructure of temporo-parietal white matter as a basis for reading ability: evidence from diffusion tensor magnetic resonance imaging. Neuron 2000; 25: 493–500. 10719902 10.1016/s0896-6273(00)80911-3

[bib128] Deutsch GK, Dougherty RF, Bammer R, Siok WT, Gabrieli JDE, Wandell B. Children’s reading performance is correlated with white matter structure measured by diffusion tensor imaging. Cortex 2005; 41: 354–363. 15871600 10.1016/s0010-9452(08)70272-7

[bib129] Richards T, Stevenson J, Crouch J, Johnson LC, Maravilla K, Stock P et al. Tract-based spatial statistics of diffusion tensor imaging in adults with dyslexia. Am J Neuroradiol 2008; 29: 1134–1139. 18467520 10.3174/ajnr.A1007 PMC2435068

[bib130] Carter JC, Lanham DC, Cutting LE, Clements-Stephens AM, Chen X, Hadzipasic M et al. A dual DTI approach to analyzing white matter in children with dyslexia. Psychiatry Res 2009; 172: 215–219. 19346108 10.1016/j.pscychresns.2008.09.005 PMC2720547

[bib131] Rollins NK, Vachha B, Srinivasan P, Chia J, Pickering J, Hughes CW et al. Simple developmental dyslexia in children: alterations in diffusion-tensor metrics of white matter tracts at 3 T. Radiology 2009; 251: 882–891. 19346513 10.1148/radiol.2513080884

[bib132] Odegard TN, Farris EA, Ring J, McColl R, Black J. Brain connectivity in non-reading impaired children and children diagnosed with developmental dyslexia. Neuropsychologia 2009; 47: 1972–1977. 19428430 10.1016/j.neuropsychologia.2009.03.009

[bib133] Rimrodt SL, Peterson DJ, Denckla MB, Kaufmann WE, Cutting LE. White matter microstructural differences linked to left perisylvian language network in children with dyslexia. Cortex 2010; 46: 739–749. 19682675 10.1016/j.cortex.2009.07.008 PMC2847658

[bib134] Vandermosten M, Boets B, Poelmans H, Sunaert S, Wouters J, Ghesquiere P. A tractography study in dyslexia: neuroanatomic correlates of orthographic, phonological and speech processing. Brain 2012; 135: 935–948. 22327793 10.1093/brain/awr363

[bib135] Hynd GW, Hall J, Novey ES, Eliopulos D, Black K, Gonzalez JJ et al. Dyslexia and corpus callosum morphology. Arch Neurol 1995; 52: 32–38. 7826273 10.1001/archneur.1995.00540250036010

[bib136] Robichon F, Habib M. Abnormal callosal morphology in male adult dyslexics: relationships to handedness and phonological abilities. Brain Lang 1998; 62: 127–146. 9570883 10.1006/brln.1997.1891

[bib137] Paulesu E, Danelli L, Berlingeri M. Reading the dyslexic brain: multiple dysfunctional routes revealed by a new meta-analysis of PET and fMRI activation studies. Front Hum Neurosci 2014; 8: 830. 25426043 10.3389/fnhum.2014.00830 PMC4227573

[bib138] Elnakib A, Soliman A, Nitzken M, Casanova MF, Gimel’farb G, El-Baz A. Magnetic resonance imaging findings for dyslexia: a review. J Biomed Nanotechnol 2014; 10: 2778–2805. 25992418 10.1166/jbn.2014.1895

[bib139] Seki A, Koeda T, Sugihara S, Kamba M, Hirata Y, Ogawa T et al. A functional magnetic resonance imaging study during sentence reading in Japanese dyslexic children. Brain Dev 2001; 23: 312–316. 11504602 10.1016/s0387-7604(01)00228-5

[bib140] Georgiewa P, Rzanny R, Gaser C, Gerhard UJ, Vieweg U, Freesmeyer D et al. Phonological processing in dyslexic children: a study combining functional imaging and event related potentials. Neurosci Lett 2002; 318: 5–8. 11786212 10.1016/s0304-3940(01)02236-4

[bib141] Karni A, Morocz IA, Bitan T, Shaul S, Kushnir T, Breznitz Z. An fMRI study of the differential effects of word presentation rates (reading acceleration) on dyslexic readers’ brain activity patterns. J Neurolinguistics 2005; 18: 197–219.

[bib142] Brambati SM, Termine C, Ruffino M, Danna M, Lanzi G, Stella G et al. Neuropsychological deficits and neural dysfunction in familial dyslexia. Brain Res 2006; 1113: 174–185. 16934234 10.1016/j.brainres.2006.06.099

[bib143] Hoeft F, Meyler A, Hernandez A, Juel C, Taylor-Hill H, Martindale JL et al. Functional and morphometric brain dissociation between dyslexia and reading ability. Proc Natl Acad Sci USA 2007; 104: 4234–4239. 17360506 10.1073/pnas.0609399104 PMC1820738

[bib144] Rimrodt SL, Clements-Stephens AM, Pugh KR, Courtney SM, Gaur P, Pekar JJ et al. Functional MRI of sentence comprehension in children with dyslexia: beyond word recognition. Cereb Cortex 2009; 19: 402–413. 18515796 10.1093/cercor/bhn092 PMC2638788

[bib145] Wimmer H, Schurz M, Sturm D, Richlan F, Klackl J, Kronbichler M et al. A dual-route perspective on poor reading in a regular orthography: an fMRI study. Cortex 46: 1284–1298. 10.1016/j.cortex.2010.06.004 PMC3073233 20650450

[bib146] Olulade OA, Flowers DL, Napoliello EM, Eden GF. Developmental differences for word processing in the ventral stream. Brain Lang 2013; 125: 134–145. 22564748 10.1016/j.bandl.2012.04.003 PMC3426643

[bib147] Olulade OA, Flowers DL, Napoliello EM, Eden GF. Dyslexic children lack word selectivity gradients in occipito-temporal and inferior frontal cortex. Neuroimage Clin 2015; 7: 742–754. 25844326 10.1016/j.nicl.2015.02.013 PMC4375638

[bib148] Saralegui I, Ontañón JM, Fernandez-Ruanova B, Garcia-Zapirain B, Basterra A, Sanz-Arigita EJ. Reading networks in children with dyslexia compared to children with ocular motility disturbances revealed by fMRI. Front Hum Neurosci 2014; 8: 936. 25477808 10.3389/fnhum.2014.00936 PMC4237045

[bib149] Shaywitz BA, Shaywitz SE, Pugh KR, Mencl WE, Fulbright RK, Skudlarski P et al. Disruption of posterior brain systems for reading in children with developmental dyslexia. Biol Psychiatry 2002; 52: 101–110. 12114001 10.1016/s0006-3223(02)01365-3

[bib150] Backes W, Vuurman E, Wennekes R, Spronk P, Wuisman M, van Engelshoven J et al. Atypical brain activation of reading processes in children with developmental dyslexia. J Child Neurol 2002; 17: 867–871. 12593457 10.1177/08830738020170121601

[bib151] Desroches AS, Cone NE, Bolger DJ, Bitan T, Burman DD, Booth JR. Children with reading difficulties show differences in brain regions associated with orthographic processing during spoken language processing. Brain Res 2010; 1356: 73–84. 20691675 10.1016/j.brainres.2010.07.097 PMC2942963

[bib152] Heim S, Grande M, Pape-Neumann J, van Ermingen M, Meffert E, Grabowska A et al. Interaction of phonological awareness and ‘magnocellular’ processing during normal and dyslexic reading: behavioural and fMRI investigations. Dyslexia 2010; 16: 258–282. 20680995 10.1002/dys.409

[bib153] Steinbrink C, Groth K, Lachmann T, Riecker A. Neural correlates of temporal auditory processing in developmental dyslexia during German vowel length discrimination: an fMRI study. Brain Lang 2012; 121: 1–11. 22377262 10.1016/j.bandl.2011.12.003

[bib154] Peyrin C, Lallier M, Démonet JF, Pernet C, Baciu M, Le Bas JF et al. Neural dissociation of phonological and visual attention span disorders in developmental dyslexia: FMRI evidence from two case reports. Brain Lang 2012; 120: 381–394. 22285025 10.1016/j.bandl.2011.12.015

[bib155] Díaz B, Hintz F, Kiebel SJ, von Kriegstein K. Dysfunction of the auditory thalamus in developmental dyslexia. Proc Natl Acad Sci USA 2012; 109: 13841–13846. 22869724 10.1073/pnas.1119828109 PMC3427071

[bib156] Liu L, Wang W, You W, Li Y, Awati N, Zhao X et al. Similar alterations in brain function for phonological and semantic processing to visual characters in Chinese dyslexia. Neuropsychologia 2012; 50: 2224–2232. 22698991 10.1016/j.neuropsychologia.2012.05.026 PMC3987122

[bib157] Olulade OA, Gilger JW, Talavage TM, Hynd GW, McAteer CI. Beyond phonological processing deficits in adult dyslexics: atypical FMRI activation patterns for spatial problem solving. Dev Neuropsychol 2012; 37: 617–635. 23066939 10.1080/87565641.2012.702826

[bib158] van Ermingen-Marbach M, Pape-Neumann J, Grande M, Grabowska A, Heim S. Distinct neural signatures of cognitive subtypes of dyslexia: effects of lexicality during phonological processing. Acta Neurobiol Exp (Wars) 2013; 73: 404–416. 24129489 10.55782/ane-2013-1947

[bib159] Hernandez N, Andersson F, Edjlali M, Hommet C, Cottier JP, Destrieux C et al. Cerebral functional asymmetry and phonological performance in dyslexic adults. Psychophysiology 2013; 50: 1226–1238. 24117474 10.1111/psyp.12141

[bib160] Kita Y, Yamamoto H, Oba K, Terasawa Y, Moriguchi Y, Uchiyama H et al. Altered brain activity for phonological manipulation in dyslexic Japanese children. Brain 2013; 136: 3696–3708. 24052613 10.1093/brain/awt248 PMC3916739

[bib161] Kronschnabel J, Brem S, Maurer U, Brandeis D. The level of audiovisual print-speech integration deficits in dyslexia. Neuropsychologia 2014; 62: 245–261. 25084224 10.1016/j.neuropsychologia.2014.07.024

[bib162] Baillieux H, Vandervliet EJM, Manto M, Parizel PM, De Deyn PP, Mariën P. Developmental dyslexia and widespread activation across the cerebellar hemispheres. Brain Lang 2009; 108: 122–132. 18986695 10.1016/j.bandl.2008.10.001

[bib163] Ruff S, Marie N, Celsis P, Cardebat D, Démonet J-F. Neural substrates of impaired categorical perception of phonemes in adult dyslexics: an fMRI study. Brain Cogn 2003; 53: 331–334. 14607175 10.1016/s0278-2626(03)00137-4

[bib164] Gaab N, Gabrieli JDE, Deutsch GK, Tallal P, Temple E. Neural correlates of rapid auditory processing are disrupted in children with developmental dyslexia and ameliorated with training: an fMRI study. Restor Neurol Neurosci 2007; 25: 295–310. 17943007

[bib165] Conway T, Heilman KM, Gopinath K, Peck K, Bauer R, Briggs RW et al. Neural substrates related to auditory working memory comparisons in dyslexia: an fMRI study. J Int Neuropsychol Soc 2008; 14: 629–639. 18577292 10.1017/S1355617708080867 PMC3010865

[bib166] Blau V, Reithler J, van Atteveldt N, Seitz J, Gerretsen P, Goebel R et al. Deviant processing of letters and speech sounds as proximate cause of reading failure: a functional magnetic resonance imaging study of dyslexic children. Brain 2010; 133: 868–879. 20061325 10.1093/brain/awp308

[bib167] Kast M, Bezzola L, Jäncke L, Meyer M. Multi- and unisensory decoding of words and nonwords result in differential brain responses in dyslexic and nondyslexic adults. Brain Lang 2011; 119: 136–148. 21641022 10.1016/j.bandl.2011.04.002

[bib168] Kovelman I, Norton ES, Christodoulou JA, Gaab N, Lieberman DA, Triantafyllou C et al. Brain basis of phonological awareness for spoken language in children and its disruption in dyslexia. Cereb Cortex 2012; 22: 754–764. 21693783 10.1093/cercor/bhr094 PMC4498147

[bib169] Dole M, Meunier F, Hoen M. Functional correlates of the speech-in-noise perception impairment in dyslexia: an MRI study. Neuropsychologia 2014; 60: 103–114. 24905286 10.1016/j.neuropsychologia.2014.05.016

[bib170] Beneventi H, Tønnessen FE, Ersland L. Dyslexic children show short-term memory deficits in phonological storage and serial rehearsal: an fMRI study. Int J Neurosci 2009; 119: 2017–2043. 19863259 10.1080/00207450903139671

[bib171] Beneventi H, Tønnessen FE, Ersland L, Hugdahl K. Executive working memory processes in dyslexia: behavioral and fMRI evidence. Scand J Psychol 2010; 51: 192–202. 20338015 10.1111/j.1467-9450.2010.00808.x

[bib172] Beneventi H, Tønnessen FE, Ersland L, Hugdahl K. Working memory deficit in dyslexia: behavioral and FMRI evidence. Int J Neurosci 2010; 120: 51–59. 20128672 10.3109/00207450903275129

[bib173] Wolf RC, Sambataro F, Lohr C, Steinbrink C, Martin C, Vasic N. Functional brain network abnormalities during verbal working memory performance in adolescents and young adults with dyslexia. Neuropsychologia 2010; 48: 309–318. 19782695 10.1016/j.neuropsychologia.2009.09.020

[bib174] Eden GF, VanMeter JW, Rumsey JM, Maisog JM, Woods RP, Zeffiro TA. Abnormal processing of visual motion in dyslexia revealed by functional brain imaging. Nature 1996; 382: 66–69. 8657305 10.1038/382066a0

[bib175] Demb JB, Boynton GM, Heeger DJ. Functional magnetic resonance imaging of early visual pathways in dyslexia. J Neurosci 1998; 18: 6939–6951. 9712663 10.1523/JNEUROSCI.18-17-06939.1998 PMC6792964

[bib176] Olulade OA, Napoliello EM, Eden GF. Abnormal visual motion processing is not a cause of dyslexia. Neuron 2013; 79: 180–190. 23746630 10.1016/j.neuron.2013.05.002 PMC3713164

[bib177] Zhang Y, Whitfield-Gabrieli S, Christodoulou JA, Gabrieli JDE. Atypical balance between occipital and fronto-parietal activation for visual shape extraction in dyslexia. PLoS One 2013; 8: e67331. 23825653 10.1371/journal.pone.0067331 PMC3692444

[bib178] Diehl JJ, Frost SJ, Sherman G, Mencl WE, Kurian A, Molfese P et al. Neural correlates of language and non-language visuospatial processing in adolescents with reading disability. Neuroimage 2014; 101: 653–666. 25067812 10.1016/j.neuroimage.2014.07.029 PMC4167780

[bib179] Peyrin C, Démonet JF, N’Guyen-Morel MA, Le Bas JF, Valdois S. Superior parietal lobule dysfunction in a homogeneous group of dyslexic children with a visual attention span disorder. Brain Lang 2011; 118: 128–138. 20739053 10.1016/j.bandl.2010.06.005

[bib180] Reilhac C, Peyrin C, Démonet J-F, Valdois S. Role of the superior parietal lobules in letter-identity processing within strings: FMRI evidence from skilled and dyslexic readers. Neuropsychologia 2013; 51: 601–612. 23270676 10.1016/j.neuropsychologia.2012.12.010

[bib181] Lobier MA, Peyrin C, Pichat C, Le Bas J-F, Valdois S. Visual processing of multiple elements in the dyslexic brain: evidence for a superior parietal dysfunction. Front Hum Neurosci 2014; 8: 479. 25071509 10.3389/fnhum.2014.00479 PMC4083222

[bib182] Raschle NM, Chang M, Gaab N. Structural brain alterations associated with dyslexia predate reading onset. Neuroimage 2011; 57: 742–749. 20884362 10.1016/j.neuroimage.2010.09.055 PMC3499031

[bib183] Vandermosten M, Vanderauwera J, Theys C, De Vos A, Vanvooren S, Sunaert S et al. A DTI tractography study in pre-readers at risk for dyslexia. Dev Cogn Neurosci 2015; 14: 8–15. 26048528 10.1016/j.dcn.2015.05.006 PMC6989819

[bib184] Dębska A, Łuniewska M, Chyl K, Banaszkiewicz A, Żelechowska A, Wypych M et al. Neural basis of phonological awareness in beginning readers with familial risk of dyslexia—results from shallow orthography. Neuroimage 2016; 132: 406–416. 26931814 10.1016/j.neuroimage.2016.02.063

[bib185] Raschle NM, Stering PL, Meissner SN, Gaab N. Altered neuronal response during rapid auditory processing and its relation to phonological processing in prereading children at familial risk for dyslexia. Cereb Cortex 2014; 24: 2489–2501. 23599167 10.1093/cercor/bht104 PMC4184369

[bib186] Pennington BF. From single to multiple deficit models of developmental disorders. Cognition 2006; 101: 385–413. 16844106 10.1016/j.cognition.2006.04.008

[bib187] Bishop D V. The interface between genetics and psychology: lessons from developmental dyslexia. Proc Biol Sci 2015; 282: 20143139. 25854887 10.1098/rspb.2014.3139 PMC4426619

[bib188] Zou L, Chen W, Shao S, Sun Z, Zhong R, Shi J et al. Genetic variant in KIAA0319, but not in DYX1C1, is associated with risk of dyslexia: an integrated meta-analysis. Am J Med Genet B Neuropsychiatr Genet 2012; 159B: 970–976. 23065966 10.1002/ajmg.b.32102

[bib189] Zhong R, Yang B, Tang H, Zou L, Song R, Zhu LQ et al. Meta-analysis of the association between DCDC2 polymorphisms and risk of dyslexia. Mol Neurobiol 2013; 47: 435–442. 23229871 10.1007/s12035-012-8381-7

[bib190] Becker J, Czamara D, Scerri TS, Ramus F, Csepe V, Talcott JB et al. Genetic analysis of dyslexia candidate genes in the European cross-linguistic NeuroDys cohort. Eur J Hum Genet 2014; 22: 675–680. 24022301 10.1038/ejhg.2013.199 PMC3992562

[bib191] Plomin R. Commentary: missing heritability, polygenic scores, and gene-environment correlation. J Child Psychol Psychiatry 2013; 54: 1147–1149. 24007418 10.1111/jcpp.12128 PMC4033839

[bib192] Maher B. Personal genomes: the case of the missing heritability. Nature 2008; 456: 18–21. 18987709 10.1038/456018a

[bib193] Yang J, Lee SH, Goddard ME, Visscher PM. GCTA: a tool for genome-wide complex trait analysis. Am J Hum Genet 2011; 88: 76–82. 21167468 10.1016/j.ajhg.2010.11.011 PMC3014363

[bib194] Poelmans G, Buitelaar JK, Pauls DL, Franke B. A theoretical molecular network for dyslexia: integrating available genetic findings. Mol Psychiatry 2011; 16: 365–382. 20956978 10.1038/mp.2010.105

[bib195] Braff DL. The importance of endophenotypes in schizophrenia research. Schizophr Res 2015; 163: 1–8. 25795083 10.1016/j.schres.2015.02.007

[bib196] Gottesman II, Gould TD. The endophenotype concept in psychiatry: etymology and strategic intentions. Am J Psychiatry 2003; 160: 636–645. 12668349 10.1176/appi.ajp.160.4.636

[bib197] Kendler KS, Neale MC. Endophenotype: a conceptual analysis. Mol Psychiatry 2010; 15: 789–797. 20142819 10.1038/mp.2010.8 PMC2909487

[bib198] Black JM, Myers CA, Hoeft F. The utility of neuroimaging studies for informing educational practice and policy in reading disorders. New Dir Child Adolesc Dev 2015; 2015: 49–56. 25732015 10.1002/cad.20086 PMC4371735

[bib199] Cope N, Eicher JD, Meng H, Gibson CJ, Hager K, Lacadie C et al. Variants in the DYX2 locus are associated with altered brain activation in reading-related brain regions in subjects with reading disability. Neuroimage 2012; 63: 148–156. 22750057 10.1016/j.neuroimage.2012.06.037 PMC3518451

[bib200] Meda SA, Gelernter J, Gruen JR, Calhoun VD, Meng H, Cope NA et al. Polymorphism of DCDC2 reveals differences in cortical morphology of healthy individuals-a preliminary voxel based morphometry study. Brain Imaging Behav 2008; 2: 21–26. 19096528 10.1007/s11682-007-9012-1 PMC2605089

[bib201] Jamadar S, Powers NR, Meda SA, Gelernter J, Gruen JR, Pearlson GD. Genetic influences of cortical gray matter in language-related regions in healthy controls and schizophrenia. Schizophr Res 2011; 129: 141–148. 21507613 10.1016/j.schres.2011.03.027 PMC3110636

[bib202] Jamadar S, Powers NR, Meda SA, Calhoun VD, Gelernter J, Gruen JR et al. Genetic influences of resting state fMRI activity in language-related brain regions in healthy controls and schizophrenia patients: a pilot study. Brain Imaging Behav 2013; 7: 15–27. 22669497 10.1007/s11682-012-9168-1 PMC4428558

[bib203] Darki F, Peyrard-Janvid M, Matsson H, Kere J, Klingberg T. Three dyslexia susceptibility genes, DYX1C1, DCDC2, and KIAA0319, affect temporo-parietal white matter structure. Biol Psychiatry 2012; 72: 671–676. 22683091 10.1016/j.biopsych.2012.05.008

[bib204] Darki F, Peyrard-Janvid M, Matsson H, Kere J, Klingberg T. DCDC2 polymorphism is associated with left temporoparietal gray and white matter structures during development. J Neurosci 2014; 34: 14455–14462. 25339756 10.1523/JNEUROSCI.1216-14.2014 PMC6608392

[bib205] Pinel P, Fauchereau F, Moreno A, Barbot A, Lathrop M, Zelenika D et al. Genetic variants of FOXP2 and KIAA0319/TTRAP/THEM2 locus are associated with altered brain activation in distinct language-related regions. J Neurosci 2012; 32: 817–825. 22262880 10.1523/JNEUROSCI.5996-10.2012 PMC6621137

[bib206] Scerri TS, Darki F, Newbury DF, Whitehouse AJO, Peyrard-Janvid M, Matsson H et al. The dyslexia candidate locus on 2p12 is associated with general cognitive ability and white matter structure. PLoS One 2012; 7: e50321. 23209710 10.1371/journal.pone.0050321 PMC3509064

[bib207] Marino C, Scifo P, Rosa PA, Della, Mascheretti S, Facoetti A, Lorusso ML et al. The DCDC2/intron 2 deletion and white matter disorganization: focus on developmental dyslexia. Cortex 2014; 57: 227–243. 24926531 10.1016/j.cortex.2014.04.016 PMC5975637

[bib208] Eicher JD, Montgomery AM, Akshoomoff N, Amaral DG, Bloss CS, Libiger O et al. Dyslexia and language impairment associated genetic markers influence cortical thickness and white matter in typically developing children. Brain Imaging Behav 2016; 10: 272–282. 25953057 10.1007/s11682-015-9392-6 PMC4639472

[bib209] Pergola G, Di Carlo P, Andriola I, Gelao B, Torretta S, Attrotto MT et al. Combined effect of genetic variants in the GluN2B coding gene (GRIN2B) on prefrontal function during working memory performance. Psychol Med 2016; 46: 1135–1150. 26690829 10.1017/S0033291715002639

[bib210] Hoogman M, Guadalupe T, Zwiers MP, Klarenbeek P, Francks C, Fisher SE. Assessing the effects of common variation in the FOXP2 gene on human brain structure. Front Hum Neurosci 2014; 8: 473. 25013396 10.3389/fnhum.2014.00473 PMC4076884

[bib211] Dennis EL, Jahanshad N, Rudie JD, Brown JA, Johnson K, McMahon KL et al. Altered structural brain connectivity in healthy carriers of the autism risk gene, CNTNAP2. Brain Connect 2011; 1: 447–459. 22500773 10.1089/brain.2011.0064 PMC3420970

[bib212] Whalley HC, O’Connell G, Sussmann JE, Peel A, Stanfield AC, Hayiou-Thomas ME et al. Genetic variation in CNTNAP2 alters brain function during linguistic processing in healthy individuals. Am J Med Genet B Neuropsychiatr Genet 2011; 156B: 941–948. 21987501 10.1002/ajmg.b.31241

[bib213] Clem von Hohenberg C, Wigand MC, Kubicki M, Leicht G, Giefling I, Karch S et al. CNTNAP2 polymorphisms and structural brain connectivity: a diffusion-tensor imaging study. J Psychiatr Res 2013; 47: 1349–1356. 23871450 10.1016/j.jpsychires.2013.07.002 PMC3780783

[bib214] Tan GCY, Doke TF, Ashburner J, Wood NW, Frackowiak RSJ. Normal variation in fronto-occipital circuitry and cerebellar structure with an autism-associated polymorphism of CNTNAP2. Neuroimage 2010; 53: 1030–1042. 20176116 10.1016/j.neuroimage.2010.02.018 PMC2941042

[bib215] Scott-Van Zeeland AA, Abrahams BS, Alvarez-Retuerto AI, Sonnenblick LI, Rudie JD, Ghahremani D et al. Altered functional connectivity in frontal lobe circuits is associated with variation in the autism risk gene CNTNAP2. Sci Transl Med 2010; 2: 56ra–80r. 10.1126/scitranslmed.3001344 PMC3065863 21048216

[bib216] Vargha-Khadem F, Watkins KE, Price CJ, Ashburner J, Alcock KJ, Connelly A et al. Neural basis of an inherited speech and language disorder. Proc Natl Acad Sci USA 1998; 95: 12695–12700. 9770548 10.1073/pnas.95.21.12695 PMC22893

[bib217] Watkins KE, Vargha-Khadem F, Ashburner J, Passingham RE, Connelly A, Friston KJ et al. MRI analysis of an inherited speech and language disorder: structural brain abnormalities. Brain 2002; 125: 465–478. 11872605 10.1093/brain/awf057

[bib218] Belton E, Salmond CH, Watkins KE, Vargha-Khadem F, Gadian DG. Bilateral brain abnormalities associated with dominantly inherited verbal and orofacial dyspraxia. Hum Brain Mapp 2003; 18: 194–200. 12599277 10.1002/hbm.10093 PMC6872113

[bib219] Liégeois F, Baldeweg T, Connelly A, Gadian DG, Mishkin M, Vargha-Khadem F. Language fMRI abnormalities associated with FOXP2 gene mutation. Nat Neurosci 2003; 6: 1230–1237. 14555953 10.1038/nn1138

[bib220] Konrad A, Vucurevic G, Musso F, Winterer G. VBM-DTI correlates of verbal intelligence: a potential link to Broca’s area. J Cogn Neurosci 2012; 24: 888–895. 22220724 10.1162/jocn_a_00187

[bib221] Zhang H, Schneider T, Wheeler-Kingshott CA, Alexander DC. NODDI: practical *in vivo* neurite orientation dispersion and density imaging of the human brain. Neuroimage 2012; 61: 1000–1016. 22484410 10.1016/j.neuroimage.2012.03.072

[bib222] Chang YS, Owen JP, Pojman NJ, Thieu T, Bukshpun P, Wakahiro MLJ et al. White matter changes of neurite density and fiber orientation dispersion during human brain maturation. PLoS One 2015; 10: e0123656. 26115451 10.1371/journal.pone.0123656 PMC4482659

[bib223] Sepehrband F, Clark KA, Ullmann JFP, Kurniawan ND, Leanage G, Reutens DC et al. Brain tissue compartment density estimated using diffusion-weighted MRI yields tissue parameters consistent with histology. Hum Brain Mapp 2015; 36: 3687–3702. 26096639 10.1002/hbm.22872 PMC4545675

[bib224] Liu B, Zhang X, Cui Y, Qin W, Tao Y, Li J et al. Polygenic risk for schizophrenia influences cortical gyrification in 2 independent general populations. Schizophr Bull 2016; doi:10.1093/schbul/sbw051. 10.1093/schbul/sbw051 PMC5463795 27169464

[bib225] Qiu L, He Y, Tang H, Zhou Y, Wang J, Zhang W et al. Genetically-mediated grey and white matter alteration in normal elderly individuals with the CLU-C allele gene. Curr Alzheimer Res 2016; http://www.ncbi.nlm.nih.gov/pubmed/27396407. 10.2174/1567205013666160703180531 PMC5112753 27396407

[bib226] Ramirez LM, Goukasian N, Porat S, Hwang KS, Eastman JA, Hurtz S et al. Common variants in ABCA7 and MS4A6A are associated with cortical and hippocampal atrophy. Neurobiol Aging 2016; 39: 82–89. 26923404 10.1016/j.neurobiolaging.2015.10.037

[bib227] Schmitt A, Rujescu D, Gawlik M, Hasan A, Hashimoto K, Iceta S et al. Consensus paper of the WFSBP Task Force on Biological Markers: criteria for biomarkers and endophenotypes of schizophrenia part II—cognition, neuroimaging and genetics. World J Biol Psychiatry 2016; 17: 406–428. 27311987 10.1080/15622975.2016.1183043

[bib228] Weiner MW, Veitch DP, Aisen PS, Beckett LA, Cairns NJ, Cedarbaum J et al. Impact of the Alzheimer’s Disease Neuroimaging Initiative, 2004 to 2014. Alzheimers Dement 2015; 11: 865–884. 26194320 10.1016/j.jalz.2015.04.005 PMC4659407

[bib229] Carroll JM, Solity J, Shapiro LR. Predicting dyslexia using prereading skills: the role of sensorimotor and cognitive abilities. J Child Psychol Psychiatry 2016; 57: 750–758. 26662375 10.1111/jcpp.12488 PMC4991277

[bib230] Gabrieli JD. Dyslexia: a new synergy between education and cognitive neuroscience. Science 2009; 325: 280–283. 19608907 10.1126/science.1171999

[bib231] Menghini D, Finzi A, Benassi M, Bolzani R, Facoetti A, Giovagnoli S et al. Different underlying neurocognitive deficits in developmental dyslexia: a comparative study. Neuropsychologia 2010; 48: 863–872. 19909762 10.1016/j.neuropsychologia.2009.11.003

[bib232] Tamboer P, Vorst HC, Oort FJ. Five describing factors of dyslexia. J Learn Disabil 2014; 49: 466–483. 25398549 10.1177/0022219414558123

[bib233] Flint J, Timpson N, Munafò M. Assessing the utility of intermediate phenotypes for genetic mapping of psychiatric disease. Trends Neurosci 2014; 37: 733–741. 25216981 10.1016/j.tins.2014.08.007 PMC4961231

[bib234] Ioannidis JPA. Why most published research findings are false. PLoS Med 2005; 2: e124. 16060722 10.1371/journal.pmed.0020124 PMC1182327

[bib235] Thompson PM, Stein JL, Medland SE, Hibar DP, Vasquez AA, Renteria ME et al. The ENIGMA Consortium: large-scale collaborative analyses of neuroimaging and genetic data. Brain Imaging Behav 2014; 8: 153–182. 24399358 10.1007/s11682-013-9269-5 PMC4008818

[bib236] Yamasue H. Using endophenotypes to examine molecules related to candidate genes as novel therapeutics: The ‘endophenotype-associated surrogate endpoint (EASE)’ concept. Neurosci Res 2015; 99: 1–7. 26055442 10.1016/j.neures.2015.05.007

[bib237] Grigorenko EL, Wood FB, Meyer MS, Hart LA, Speed WC, Shuster A et al. Susceptibility loci for distinct components of developmental dyslexia on chromosomes 6 and 15. Am J Hum Genet 1997; 60: 27–39. 8981944 PMC1712535

[bib238] Addis L, Friederici AD, Kotz SA, Sabisch B, Barry J, Richter N et al. A locus for an auditory processing deficit and language impairment in an extended pedigree maps to 12p13.31-q14.3. Genes Brain Behav 2010; 9: 545–561. 20345892 10.1111/j.1601-183X.2010.00583.x PMC2948670

[bib239] Shao S, Niu Y, Zhang X, Kong R, Wang J, Liu L et al. Opposite associations between individual KIAA0319 polymorphisms and developmental dyslexia risk across populations: a stratified meta-analysis by the study population. Sci Rep 2016; 6: 30454. 27464509 10.1038/srep30454 PMC4964335

[bib240] Mann A, Machado NM, Liu N, Mazin A-H, Silver K, Afzal KI. A multidisciplinary approach to the treatment of anti-NMDA-receptor antibody encephalitis: a case and review of the literature. J Neuropsychiatry Clin Neurosci 2012; 24: 247–254. 22772674 10.1176/appi.neuropsych.11070151

[bib241] Homberg JR, Kyzar EJ, Stewart AM, Nguyen M, Poudel MK, Echevarria DJ et al. Improving treatment of neurodevelopmental disorders: recommendations based on preclinical studies. Expert Opin Drug Discov 2016; 11: 11–25. 26558752 10.1517/17460441.2016.1115834

[bib242] Plak RD, Kegel CAT, Bus AG. Genetic differential susceptibility in literacy-delayed children: a randomized controlled trial on emergent literacy in kindergarten. Dev Psychopathol 2015; 27: 69–79. 25640831 10.1017/S0954579414001308

[bib243] Glahn DC, Curran JE, Winkler AM, Carless MA, Kent JW Jr, Charlesworth JC et al. High dimensional endophenotype ranking in the search for major depression risk genes. Biol Psychiatry 2012; 71: 6–14. 21982424 10.1016/j.biopsych.2011.08.022 PMC3230692

[bib244] Dima D, Breen G. Polygenic risk scores in imaging genetics: usefulness and applications. J Psychopharmacol 2015; 29: 867–871. 25944849 10.1177/0269881115584470

