# Supplementary material for: Neurogenetics of developmental dyslexia: from genes to behavior through brain neuroimaging and cognitive and sensorial mechanisms
Source: Transl Psychiatry. 2017 Jan 3;7(1):e987–. doi: 10.1038/tp.2016.240 (PMC5545717; doi:10.1038/tp.2016.240)
Supplement: Supplementary Table 1 [file tp2016240x1.doc]

**Supplementary Table 1. Overview of the findings obtained with structural MR techniques (VBM, DTI) children with and without DD.**

| **VBM** | | | | |
| --- | --- | --- | --- | --- |
| **Study** | **Subjects** | **MR Field** | **Analysis** | **Results** |
| Eliez et al. 2000^1^ | 16 DD vs 14 NR* | 1.5T | VBM BrainImage | Reduced GMV in the left temporal lobe |
| Brown et al. 2001^2^ | 16 DD vs 14 NR* | 1.5T | VBM SPM99 | Reduction of GMV in left temporal lobe and bilateral temporo-parietal-occipital junction, frontal lobe, caudate, thalamus and cerebellum |
| Brambati et al. 2004^3^ | 10 DD vs 11 NR* | 1.5T | VBM SPM2 | Alterations of GMV in bilateral planum temporale, inferior temporal cortex, cerebellar nuclei |
| Eckert et al. 2005^4^ | 13 DD vs 13 NR* | 1.5T | VBM SPM2 | Alterations of GMV in the left temporo-parietal region, bilateral lingual gyrus, left inferior parietal lobule and cerebellum. |
| Silani et al. 2005^5^ | 10+11+11 DD vs 9+12+11 NR* | 1.5T/2T | VBM SPM5 | Alterations of GMV in left middle and inferior temporal gyri and WMV in the left arcuate fasciculus. |
| Vinckenbosch et al. 2005^6^ | 14 DD vs 10 NR | 1.5T | VBM BrainImage + SPM99 | Reduced GMV in middle and inferior temporal gyri. Increased GM in the precentral gyri bilaterally. |
| Hoeft et al. 2007^7^ | 23 DD vs 19 NR*+12 NR° | 3T | VBM SPM2 | Reduced GMV in the bilateral inferior parietal lobule, superior temporal, precentral and insula regions. |
| Kronbichler et al. 2008^8^ | 13 DD vs 15 NR* | 1.5T | VBM SPM2 | Reduced GMV in bilateral fusiform gyrus, anterior cerebellum and right supramaginal gyruss. |
| Siok et al. 2008^9^ | 16 DD vs 16 NR* | 2T | VBM SPM2 | Reduced WMV in left middle frontal gyrus. |
| Steinbrink et al. 2008^10^ | 8 DD vs 8 NR* | 3T | VBM/Voxelwise DTI | Alterations of GMV n bilateral superior temporal gyri. Decreased FA in bilateral fronto-temporal and left temporo-parietal WM (inferior and superior longitudinal fasciculus). FA correlated with reading performances. |
| Pernet et al. 2009^11^ | 38 DD vs 39 NR* | 1.5T | VBM SPM5 | No global differences. Correlations of GMV with reading performances in the left inferior frontal gyrus, left superior temporal gyrus, fusiform gyri and cerebellum. |
| Raschle et al., 2011^12^ | 10 FRD^+^ vs 10 FRD^-^ | 3T | VBM SPM5 | PR-FH^+^ had reduced volume in left occipitotemporal area, left and right temporo-parietal regions, left fusiform gyrus, right lingual gyrus |
| Dole et al. 2013^13^ | 14 DD vs 14 NR* | 3T | VBM SPM8 | Alterations of GMV in the superior temporal gyrus, superior temporal sulcus. |
| Liu et al. 2013^14^ | 18 DD vs 18 NR* | 3T | VBM SPM5 | Reduced GMV in right inferior occipital gyrus, left inferior frontal gyrus. Reduced WMV in left precentral gyrus. GMV of prefrontal cortices correlated with GMV of parahippocampal gyrus. |
| Krafnick et al. 2014^15^ | 15 DD vs 15 NR*+15 NR° | 3T | VBM SPM8 | Reduced GMV in left superior temporal sulcus compared to controls, reduced GMV in right precentral gyrus compared to skill-matched controls |
| Stoodley 2014^16^ | Meta-Analysis of cerebellum in ASD, ADHD and DD | - | VBM | Different cerebellar areas are involved in ASD, ADHD and DD. |
| Tamboer et al. 2015^17^ | 37 DD vs 57 NR* | 3T | VBM FSL | No global differences. Correlations of GMV in bilateral caudate nucleus and reading performances. |
| Xia et al. 2016^18^ | 24 DD vs 12 NR*+12 NR° | 3T | VBM SPM8 | Reduced GMV in the left temporo-parietal cortex, middle temporal gyrus, superior occipital gyrus. Reduced WMV in bilateral parieto-occipital regions. |
| **DTI** | | | | |
| **Study** | **Subjects** | **Field** | **Analysis** | **Results** |
| Klingberg et al. 2000^19^ | 6 DD vs 11 NR | 1.5T | VBA with SPM96 | Decreased FA in temporo-parietal WM, and was correlated with reading scores in the same regions of the left hemisphere. |
| Deutsch et al. 2005^20^ | 7 DD vs 7 NR* | 1.5T | VBA of FA, CI and ADC with SPM99 | FA and CI correlated with reading performances in the left temporo-parietal pathways. |
| Niogi & McCandlliss 2006^21^ | 11 DD vs 20 NR* | 1.5T | ROI analysis | FA of the left temporo-parietal WM correlated with reading scores, FA of the frontal WM correlated with working memory. |
| Frye et al. 2008^22^ | 9 DD vs 18 NR* | 3T | ROI analysis (only within CC) | Higher FA and AD in the Splenium. Ortographic performances negatively correlated with FA and AD for both NR and DD, PA positively correlated only for NR but not for DD. |
| Richards et al. 2008^23^ | 14 DD vs 7 NR | 1.5T | VBA based on TBSS | Reduced FA in frontal lobe, temporal lobe, occipital lobe, parietal lobe. |
| Carter et al. 2009^24^ | 7 DD vs 6 NR* | 1.5T | VBA+ROI of FA | Reduced FA in left superior longitudinal fasciculus and abnormal orientation. |
| Odegard et al. 2009^25^ | 10 DD vs 7 NR* | 3T | VBA based on TBSS | Negative correlations of FA with reading scores in the left posterior CC, positive correlations in the left superior corona radiata. |
| Rollins et al. 2009^26^ | 19 DD vs 18 NR* | 3T | ROI analysis | FA higher in inferior fronto occipital and inferior longitudinal fasciculi up to 11 years, lower after |
| Rimrodt et al. 2010^27^ | 14 DD vs 17 NR* | 3T | VBA of principal direction and ROI (tractography) of FA | Reduced FA in the left inferior frontal gyrus and left temporo-parietal WM. FA correlated positively with reading speed in a posterior circuit. |
| Hasan et al. 2012^28^ | 24 DD vs 11 NR* | 3T | ROI analysis (only within CC) | Higher FA in the posterior part of CC, with reduced RD. MD/FA of the posterior part of CC correlated positively/negatively with the reading performances. |
| Vandermosten et al. 2012^29^ | 20 DD vs 20 NR* | 3T | ROI analysis (from tractography) | Reduced FA in the left arcuate fasciculus. Correlation between FA and reading performances in the left inferior fronto-occipital fasciculus. |
| Vandermosten et al. 2013^30^ | 20 DD vs 20 NR* | 3T | DTI and ASSR correlations | Reduced WM lateralization in the posterior superior temporal gyrus and arcuate fasciculus. WM lateralization of the first and WM values of the splenium related to interhemispheric coherence oppositely to NR. |
| Richards et al. 2015^31^ | 17 DD vs 14 DS vs 19 NR | 3T | ROI correlations with fMRI | Differences in white matter integrity, functional connectivity and WM-GM correlations. |
| Vandermosten et al., 2015^32^ | 36 FRD^+^ vs 35 FRD^-^ | 3T | Tract based analysis with pre-defined ROIs with ExploreDTI | In all subjects, correlation between FA and phonological awareness in bilateral inferior fronto-occipital fasciculus and left arcuate fasciculus, and between rapid automatised naming and FA in right inferior fronto-occipital fasciculus.  FA was significantly lower in FRD+ pre-readers than in FRD− pre-readers for left inferior fronto-occipital fasciculus. |

DD=developmental dyslexia; NR=normal readers; GM=Gray Matter; WM=White Matter; GMV=Gray Matter Volume; WMV=White Matter Volume; ROI=Region Of Interest; CC=Corpus Callosum; ASD=Autism Spectrum Disorder; ADHD=Attention Deficit Hyperactivity Disorder; ASSR=Auditory Steady State Response; VBA=Voxel Based Analysis; FA=fractional anisotropy.

FRD^+^ = pre-readers with a family risk for DD; PR-FH^-^ = pre-readers without a family risk for DD.

* age-matched normal readers

° reading level normal readers

**References**

1 Eliez S, Rumsey JM, Giedd JN, Schmitt JE, Patwardhan AJ, Reiss AL. Morphological alteration of temporal lobe gray matter in dyslexia: an MRI study. *J Child Psychol Psychiatry* 2000; **41**: 637–644.

2 Brown WE, Eliez S, Menon V, Rumsey JM, White CD, Reiss AL. Preliminary evidence of widespread morphological variations of the brain in dyslexia. *Neurology* 2001; **56**: 781–783.

3 Brambati SM, Termine C, Ruffino M, Stella G, Fazio F, Cappa SF *et al.* Regional reductions of gray matter volume in familial dyslexia. *Neurology* 2004; **63**: 742–5.

4 Eckert MA, Leonard CM, Wilke M, Eckert M, Richards T, Richards A *et al.* Anatomical signatures of dyslexia in children: unique information from manual and voxel based morphometry brain measures. *Cortex* 2005; **41**: 304–15.

5 Silani G, Frith U, Demonet J-F, Fazio F, Perani D, Price C *et al.* Brain abnormalities underlying altered activation in dyslexia: a voxel based morphometry study. *Brain* 2005; **128**: 2453–61.

6 Vinckenbosch E, Robichon F, Eliez S. Gray matter alteration in dyslexia: converging evidence from volumetric and voxel-by-voxel MRI analyses. *Neuropsychologia* 2005; **43**: 324–31.

7 Hoeft F, Ueno T, Reiss AL, Meyler A, Whitfield-Gabrieli S, Glover GH *et al.* Prediction of children’s reading skills using behavioral, functional, and structural neuroimaging measures. *Behav Neurosci* 2007; **121**: 602–13.

8 Kronbichler M, Wimmer H, Staffen W, Hutzler F, Mair A, Ladurner G. Developmental dyslexia: gray matter abnormalities in the occipitotemporal cortex. *Hum Brain Mapp* 2008; **29**: 613–25.

9 Siok WT, Niu Z, Jin Z, Perfetti CA, Tan LH. A structural-functional basis for dyslexia in the cortex of Chinese readers. *Proc Natl Acad Sci U S A* 2008; **105**: 5561–6.

10 Steinbrink C, Vogt K, Kastrup A, Müller H-P, Juengling FD, Kassubek J *et al.* The contribution of white and gray matter differences to developmental dyslexia: insights from DTI and VBM at 3.0 T. *Neuropsychologia* 2008; **46**: 3170–8.

11 Pernet CR, Poline JB, Demonet JF, Rousselet GA. Brain classification reveals the right cerebellum as the best biomarker of dyslexia. *BMC Neurosci* 2009; **10**: 67.

12 Raschle NM, Chang M, Gaab N. Structural brain alterations associated with dyslexia predate reading onset. *Neuroimage* 2011; **57**: 742–9.

13 Dole M, Meunier F, Hoen M. Gray and white matter distribution in dyslexia: a VBM study of superior temporal gyrus asymmetry. *PLoS One* 2013; **8**: e76823.

14 Liu L, You W, Wang W, Guo X, Peng D, Booth J. Altered brain structure in Chinese dyslexic children. *Neuropsychologia* 2013; **51**: 1169–76.

15 Krafnick AJ, Flowers DL, Luetje MM, Napoliello EM, Eden GF. An investigation into the origin of anatomical differences in dyslexia. *J Neurosci* 2014; **34**: 901–8.

16 Stoodley CJ. Distinct regions of the cerebellum show gray matter decreases in autism, ADHD, and developmental dyslexia. *Front Syst Neurosci* 2014; **8**: 92.

17 Tamboer P, Scholte HS, Vorst HCM. Dyslexia and voxel-based morphometry: correlations between five behavioural measures of dyslexia and gray and white matter volumes. *Ann Dyslexia* 2015; **65**: 121–41.

18 Xia Z, Hoeft F, Zhang L, Shu H. Neuroanatomical anomalies of dyslexia: Disambiguating the effects of disorder, performance, and maturation. *Neuropsychologia* 2016; **81**: 68–78.

19 Klingberg T, Hedehus M, Temple E, Salz T, Gabrieli JD, Moseley ME *et al.* Microstructure of temporo-parietal white matter as a basis for reading ability: evidence from diffusion tensor magnetic resonance imaging. *Neuron* 2000; **25**: 493–500.

20 Deutsch GK, Dougherty RF, Bammer R, Siok WT, Gabrieli JDE, Wandell B. Children’s reading performance is correlated with white matter structure measured by diffusion tensor imaging. *Cortex* 2005; **41**: 354–63.

21 Niogi SN, McCandliss BD. Left lateralized white matter microstructure accounts for individual differences in reading ability and disability. *Neuropsychologia* 2006; **44**: 2178–88.

22 Frye RE, Hasan K, Xue L, Strickland D, Malmberg B, Liederman J *et al.* Splenium microstructure is related to two dimensions of reading skill. *Neuroreport* 2008; **19**: 1627–31.

23 Richards T, Stevenson J, Crouch J, Johnson LC, Maravilla K, Stock P *et al.* Tract-based spatial statistics of diffusion tensor imaging in adults with dyslexia. *AJNR Am J Neuroradiol* 2008; **29**: 1134–9.

24 Carter JC, Lanham DC, Cutting LE, Clements-Stephens AM, Chen X, Hadzipasic M *et al.* A dual DTI approach to analyzing white matter in children with dyslexia. *Psychiatry Res* 2009; **172**: 215–9.

25 Odegard TN, Farris EA, Ring J, McColl R, Black J. Brain connectivity in non-reading impaired children and children diagnosed with developmental dyslexia. *Neuropsychologia* 2009; **47**: 1972–7.

26 Rollins NK, Vachha B, Srinivasan P, Chia J, Pickering J, Hughes CW *et al.* Simple developmental dyslexia in children: alterations in diffusion-tensor metrics of white matter tracts at 3 T. *Radiology* 2009; **251**: 882–91.

27 Rimrodt SL, Peterson DJ, Denckla MB, Kaufmann WE, Cutting LE. White matter microstructural differences linked to left perisylvian language network in children with dyslexia. *Cortex* 2010; **46**: 739–49.

28 Hasan KM, Molfese DL, Walimuni IS, Stuebing KK, Papanicolaou AC, Narayana PA *et al.* Diffusion tensor quantification and cognitive correlates of the macrostructure and microstructure of the corpus callosum in typically developing and dyslexic children. *NMR Biomed* 2012; **25**: 1263–70.

29 Vandermosten M, Boets B, Poelmans H, Sunaert S, Wouters J, Ghesquiere P. A tractography study in dyslexia: neuroanatomic correlates of orthographic, phonological and speech processing. *Brain* 2012; **135**: 935–948.

30 Vandermosten M, Poelmans H, Sunaert S, Ghesquière P, Wouters J. White matter lateralization and interhemispheric coherence to auditory modulations in normal reading and dyslexic adults. *Neuropsychologia* 2013; **51**: 2087–99.

31 Richards TL, Grabowski TJ, Boord P, Yagle K, Askren M, Mestre Z *et al.* Contrasting brain patterns of writing-related DTI parameters, fMRI connectivity, and DTI-fMRI connectivity correlations in children with and without dysgraphia or dyslexia. *NeuroImage Clin* 2015; **8**: 408–21.

32 Vandermosten M, Vanderauwera J, Theys C, De Vos A, Vanvooren S, Sunaert S *et al.* A DTI tractography study in pre-readers at risk for dyslexia. *Dev Cogn Neurosci* 2015; **14**: 8–15.
