# Supplementary material for: Neurogenetics of developmental dyslexia: from genes to behavior through brain neuroimaging and cognitive and sensorial mechanisms
Source: Transl Psychiatry. 2017 Jan 3;7(1):e987–. doi: 10.1038/tp.2016.240 (PMC5545717; doi:10.1038/tp.2016.240)
Supplement: Supplementary Table 2 [file tp2016240x2.doc]

**Supplementary Table 2. Overview of the findings obtained with functional MRI on children with and without DD with tasks targeting the reading circuit.**

| **fMRI** | | | | | |
| --- | --- | --- | --- | --- | --- |
| **Study** | **Subjects** | **Field** | **Task** | **Analysis** | **Results** |
| Peyrin et al. 2011^1^ | 12 DD vs 12 NR* | 3T | attentional task | Voxelwise and ROIs with SPM2 | Reduced activation in bilateral parietal and temporal areas during flanked processing. Lack of activation in left precuneus, superior parietal lobule. |
| Reilhac et al. 2013^2^ | 12 DD vs 12 NR* | 3T |  | ROI based with SPM8 | Reduced activation in the left superior parietal lobule and left ventral occipito-temporal cortex. |
| Lobier et al. 2014^3^ | 12 DD vs 12 NR* | 3T |  | Voxelwise with SPM5 + ROIs (PickAtlas) | Reduced right superior parietal lobule activation. With ROIs reduced activation in bilateral superior parietal lobule and occipito temporal cortex. Correlation between these two areas only for DD. |
| Temple et al. 2000^4^ | 8 DD vs 10 NR* | 1.5T | auditory task | Voxelwise and ROIs with SPM96 | Lack of differential frontal response between slow and rapid processing. After remediation increased activation in the left prefrontal cortex was observed. |
| Ruff et al. 2003^5^ | 12 DD vs 14 NR* | 1.5T |  | Voxelwise with SPM99 | Lack of activation in right inferior frontal gyrus and right superior cingulate cortex. |
| Gaab et al. 2007^6^ | 22 DD vs 23 NR* | 3T |  | Voxelwise with SPM99 | No differential response between slow and rapid transitions of sounds in the left prefrontal cortex. Significant activations after remediation. |
| Conway et al. 2008^7^ | 11 DD vs 11 NR* | 3T |  | Voxelwise with AFNI | Greater activity in the left posterior superior temporal lobe, inferior parietal regions. Greater primary auditory cortex activity. |
| Blau et al. 2009^8^ | 13 DD vs 13 NR* | 3T |  | Voxelwise with BrainVoyager | Reduced activation of superior temporal cortex during the integration of letters and speech sounds. |
| Blau et al. 2010^9^ | 18 DD vs 16 NR* | 3T |  | Voxelwise with BrainVoyager | Reduced response to processing of speech sounds in the anterior superior temporal gyrus, planum temporale, heschl sulcus and superior temporal sulcus. |
| Heim et al. 2010^10^ | 57 DD vs 60 NR* | 1.5T |  | Voxelwise with SPM | Reduced activation in the auditory cortex. Increased right frontal activation except during phonological discrimination, when the right frontal cortex activation is reduced. |
| Kast et al. 2011^11^ | 12 DD vs 13 NR* | 3T |  | Voxelwise with SPM5 and ROIs | Reduced activation in the left supramarginal gyrus, right superior temporal sulcus. Reduced responses to auditory signals and enhanced response to visual and combined stimuli in the right anterior insula. |
| Kovelman et al. 2012^12^ | 12 DD vs 17 NR* + 12 NR° | 3T |  | Voxelwise with SPM2 | Lack of activation of the left dorso-lateral prefrontal cortex. |
| Steibrink et al. 2012^13^ | 17 DD vs 16 NR* | 3T |  | Voxelwise with SPM5 | decreased activation of the insula and left inferior frontal gyrus. |
| Raschle et al. 2014^14^ | 14 FRD^-^ vs 14 FRD^+^ | 3T |  | ROI based with SPM5 | Whole-brain analysis revealed that FRD- showed hyperactivation in the IFG and MFG. A direct comparison between children with and without a familial risk for DD (FRD+<FRD−) revealed differences in left-hemispheric frontal brain areas, in the left cerebellum/FG and right MFG. |
| Shaywitz et al. 1998^15^ | 29 DD vs 32 NR* | 1.5T | phonological task | ROIs based with SPM96 | Underactivation in Wernicke area, angular gyrus and striate cortex. Greater activation in inferior frontal gyrus. |
| Backes et al. 2002^16^ | 8 DD vs 8 NR* | 1.5T |  | Voxelwise with SPM99 | Enhanced activation in the left extrastriate cortex. Missing activation of the left prefrontal cortex. Reduced activation of the temporal and prefrontal cortex. |
| Shaywitz et al. 2002^17^ | 70 DD vs 74 NR* | 1.5T |  | Voxelwise and ROIs with custom software | Altered activations in the parieto-temporal and occipito-temporal cortex. Correlation with reading skill in the left occipito-temporal region. Greater activation in bilateral inferior frontal gyri in older DD compared to younger DD. |
| Siok et al. 2008^18^ | 16 DD vs 16 NR* | 2T |  | Voxelwise and ROIs with SPM2 | Reduced activation in the left middle frontal gyrus. Correlations between GMV and activation in the same area. |
| Desroches et al. 2010^19^ | 12 DD vs 12 NR* | 1.5T |  | Voxelwise with SPM2 | Lack of activation of the left fusiform cortex. Positive correlation between the activation of the left fusiform and non-word reading. |
| Diaz et al. 2012^20^ | 14 DD vs 14 NR* | 3T |  | ROI based with SPM8 | Altered activation of the medial geniculate body. This also correlated with diagnostic scores. |
| Liu et al. 2012^21^ | 16 DD vs 16 NR* | 3T |  | Voxelwise with SPM5 | Reduced activation in right visual and left occipito-temporal cortex. Reduced activation in left inferior frontal gyrus that was correlated with bilateral visuo ortographic regions. |
| Olulade et al. 2012^22^ | 9 DD vs 12 NR* | 3T |  | Voxelwise with SPM8 | Underactivation in the left fusiform gyrus during word task. During non-words under activation of left middle temporal gyrus, bilateral middle frontal gyrus, inferior parietal lobe, post-central gyrus. |
| Peyrin et al. 2012^23^ | 2 DD vs 14 NR* | 3T |  | Voxelwise and ROIs with SPM2 | Comparison of two single cases to controls, shows different patterns to highlight heterogeneity of DD |
| Steibrink et al. 2012^13^ | 17 DD vs 16 NR* | 3T |  | Voxelwise with SPM5 | No significant differences |
| Hernandez et al. 2013^24^ | 15 DD vs 16 NR* | 1.5T |  | Voxelwise SPM5, Lateralization with AAL | Asymmetry in inferior frontal gyrus correlated with performance during the phonological processing. |
| Kita et al. 2013^25^ | 14 DD vs 15 NR* + 30 adult NR | 1.5T |  | Voxelwise with SPM8 | Greater activity in the basal ganglia, reduced activity in the left superior temporal gyrus. |
| van Ermingen-Marbach et al. 2013^26^ | 15 DD^1^ vs 17 DD^2^ vs 10 NR* | 3T |  | Voxelwise with SPM5 | DD with phonological awareness and rapid naming deficits showed strong activation in left areas 44 and 45. DD with rapid naming deficits only showed activations in right areas 44 and 45. |
| Dole et al. 2014^27^ | 14 DD vs 14 NR* | 3T |  | Voxelwise with SPM8 | Increased activation in the right superior temporal gyrus in binaural configuration. |
| Kronschnabel et al. 2014^28^ | 13 DD vs 22 NR* | 3T |  | Voxelwise with SPM8 | Altered activations in left inferior frontal ad angular gyri, bilateral inferior and superior temporal cortex. |
| Debska et al., 2016^29^ | 17 FRD^+^ vs 13 FRD^-^ ^(3)^ + 45 FRD^+^ vs 27 FRD^- (4)^ | 3T |  | Voxelwise with SPM8 | FHD+ showed hypoactivation in the bilateral temporal, tempo-parietal and inferior temporal–occipital regions, as well as the bilateral IFG and MFG. Subcortically, hypoactivation was found in the bilateral thalami, caudate, and right putamen in FHD+. A main effect of the children's grade was present only in the left IFG, where reduced activation was shown in first-graders. The FG and the right middle frontal and postcentral gyri displayed an interaction between familial risk and grade. |
| Seki et al. 2001^30^ | 5 DD vs 5 NR* | 1.5T | reading-related task | Voxelwise with MEDx | Reduced activation of the middle temporal gyrus. Different activations within the single DD subjects, including bilateral occipital cortex, precentral gyrus. |
| Georgiewa et al. 2002^31^ | 9 DD vs 8 NR* | 1.5T |  | Voxelwise with SPM96 | Increased activation in the left inferior frontal gyrus. |
| Karni et al. 2005^32^ | 8 DD vs 8 NR* | 2T |  | Voxelwise with SPM99 | No differences with slow rate with words. With non-words activation of the left inferior frontal gyrus only in DD. Fast reading led to lower activations in visual areas. |
| Brambati et al. 2006^33^ | 13 DD vs 11 NR* | 1.5T |  | Voxelwise with SPM2 | Lack of activation in the posterior areas of the reading network. |
| Hoeft et al. 2007^34^ | 23 DD vs 30 NR* | 3T |  | Voxelwise and ROIs with SPM2 | Compared to age-matched controls DD showed Reduced activation in left parietal and bilateral fusiform cortex. Increased activation in left inferior and middle frontal gyri, left caudate and right thalamus. Compared to skill-related controls, no increased activations. |
| Rimrodt et al. 2009^35^ | 14 DD vs 15 NR* | 1.5T |  | Voxelwise with SPM2 | Increased activation in the left middle and superior temporal gyri, bilateral insula, right cingulate gyrus, right superior frontal gyrus, right parietal lobe. Correlations between word and reading fluency and activations in left occipito-temporal cortex. Worse performances associated with greater activations in right supramarginal and superior temporal gyri. |
| Wimmer et al. 2010^36^ | 20 DD vs 19 NR* | 1.5T |  | Voxelwise and ROIs with SPM2 | Underactivation in the left occipito-temporal region, left inferior parietal region and left inferior frontal region. Increased activation in visual occipital regions. |
| Olulade et al. 2013^37^ | 15 Adults NR vs 11 Children NR | 3T |  | ROIs with SPM8 | Differences between children and adults in the anterior left occipito-temporal cortex. |
| Saralegui et al. 2014^38^ | 19 DD vs 19 NR* vs 17 vision impaired | 3T |  | Voxelwise and ROIs with FSL | Less activation in right Broca's areas. Increased activation in bilateral medial temporal gyrus. Correlations of ROIs with scores. |
| Olulade et al. 2015^39^ | 16 DD vs 12 NR* | 3T |  | Voxelwise and ROIs with SPM8 | reduced activation in left fusiform gyrus and right middle frontal gyrus during word reading. Lower activation in the left superior parietal lobule and bilateral middle occipital regions during false-fonts. |
| Baillieux et al. 2009^40^ | 15 DD vs 7 NR* | 1.5T | semantic task | Voxelwise with BrainVoyager | Different pattern of activation. Widespread and diffuse activations in frontal, parietal, temporal, occipital and cerebellar regions. Different functioning of the cerebellar areas during semantic association. |
| Eden et al. 1996^41^ | 6 DD vs 8 NR* | 1.5T | visual task | Voxelwise in house | Lack of activation in the extrastriate visual area. No differences in stationary patterns. |
| Pugh et al. 1997^40^ | 29 DD vs 32 NR* | 1.5T |  | ROI based covariance / connectivity | Reduced functional connectivity in the left hemisphere with tasks demanding phonological assembly. |
| Demb et al. 1998^41^ | 5 DD vs 5 NR* | 1.5T |  | Voxelwise in house | Reduced brain activity in primary visual cortex and extrastriate areas. Strong correlation between brain activity, speed discrimination thresholds and reading speed. |
| Backes et al. 2002^16^ | 8 DD vs 8 NR* | 1.5T |  | Voxelwise with SPM99 | Enhanced activation in the left extrastriate cortex. Missing activation of the left prefrontal cortex. |
| Blau et al. 2009^8^ | 13 DD vs 13 NR* | 3T |  | Voxelwise with BrainVoyager | Reduced activation of superior temporal cortex during the integration of letters and speech sounds. Correlations of the reduced activation with auditory processing that predicts performance of phonological tasks. |
| Blau et al. 2010^9^ | 18 DD vs 16 NR* | 3T |  | Voxelwise with BrainVoyager | Reduced unisensory response to letters in the fusiform gyrus. |
| Heim et al. 2010^10^ | 57 DD vs 60 NR* | 1.5T |  | Voxelwise with SPM | Reduced activation in the visual cortex during motion detection. Increased right frontal activation. |
| Olulade et al. 2012^22^ | 9 DD vs 12 NR* | 3T |  | Voxelwise with SPM8 | During spatial rotation task DD showed under-activation in superior parietal lobe and precuneus, middle and pre-frontal cortex. During spatial non-rotation underactivation in occipital and motor areas. greater activation in right fusiform gyrus and right medial and superior frontal gyri. |
| Olulade et al. 2013^42^ | 14 DD vs 14 NR* + 12 DD vs 12 NR° | 3T |  | Voxelwise and ROIs with SPM8 | Underactivation in region V5/MT |
| Zhang et al. 2013^43^ | 11 DD vs 13 NR* | 3T |  | Voxelwise and ROIs with SPM5 | Decreased activation in cuneus and calcarine. Increased activation in frontal (IFG, Pre-central, MFG, SFG) and parietal regions (IPL, SMG, Post-central). |
| Diehl et al. 2014^44^ | 11 DD vs 10 NR* | 3T |  | Voxelwise BioImageSuite SPM2 | Lower activation in right fusiform gyrus, bilateral occipital gyrus, left fusiform gyrus, left putamen and insula, left inferior parietal lobule, bilateral prefrontal cortex. |
| Beneventi et al. 2009^45^ | 11 DD vs 13 NR* | 1.5T | working memory task | Voxelwise with SPM2 | In the letter probe task reduced activation in the left precentral gyrus. In the sequence probe task reduced activation in the prefrontal cortex and the superior parietal cortex. |
| Beneventi et al. 2010^46^ | 11 DD vs 13 NR* | 1.5T |  | Voxelwise with SPM2 | Reduced fMRI activation in the left superior parietal lobule and right inferior prefrontal gyrus. Missing increased activation in working memory areas with increased memory load. |
| Beneventi et al. 2010^47^ | 12 DD vs 14 NR* | 1.5T |  | Voxelwise with SPM2 | Reduced fMRI activation in the prefrontal and parietal cortex and cerebellum compared to controls. |
| Wolf et al. 2010^48^ | 12 DD vs 13 NR* | 3T |  | Voxelwise and connectivity with SPM5 | Increased functional connectivity in left prefrontal and inferior parietal regions. Decreased connectivity including bilateral dorso-lateral prefrontal and posterior parietal regions. Increased connectivity in the left angular gyrus, left hippocampal cortex and right thalamus. Connectivity of hippocampal cortex and thalamus correlated with task accuracy and number of errors during spelling test. |

DD=developmental dyslexia; NR=normal readers; ROI=Region Of Interest; IFG=Inferior Frontal Gyrus; MFG=Middle Frontal Gyrus; FG=fusiform gyrus; SFG=Superior Frontal Gyrus; IPL=Inferior Parietal Lobe; SMG=Supra Marginal Gyrus.

FRD^+^ = pre-readers with a family risk for DD; PR-FH^-^ = pre-readers without a family risk for DD.

* age-matched normal readers.

° reading level normal readers.

^1^ phonological and rapid-automatized naming deficits.

^2^ rapid-automatized naming deficit.

^3^ kindergarten pupils.

^4^ first grade pupils.

**References**

1 Peyrin C, Démonet JF, N’Guyen-Morel MA, Le Bas JF, Valdois S. Superior parietal lobule dysfunction in a homogeneous group of dyslexic children with a visual attention span disorder. *Brain Lang* 2011; **118**: 128–38.

2 Reilhac C, Peyrin C, Démonet J-F, Valdois S. Role of the superior parietal lobules in letter-identity processing within strings: FMRI evidence from skilled and dyslexic readers. *Neuropsychologia* 2013; **51**: 601–12.

3 Lobier MA, Peyrin C, Pichat C, Le Bas J-F, Valdois S. Visual processing of multiple elements in the dyslexic brain: evidence for a superior parietal dysfunction. *Front Hum Neurosci* 2014; **8**: 479.

4 Klingberg T, Hedehus M, Temple E, Salz T, Gabrieli JD, Moseley ME *et al.* Microstructure of temporo-parietal white matter as a basis for reading ability: evidence from diffusion tensor magnetic resonance imaging. *Neuron* 2000; **25**: 493–500.

5 Ruff S, Marie N, Celsis P, Cardebat D, Démonet J-F. Neural substrates of impaired categorical perception of phonemes in adult dyslexics: an fMRI study. *Brain Cogn* 2003; **53**: 331–4.

6 Gaab N, Gabrieli JDE, Deutsch GK, Tallal P, Temple E. Neural correlates of rapid auditory processing are disrupted in children with developmental dyslexia and ameliorated with training: an fMRI study. *Restor Neurol Neurosci* 2007; **25**: 295–310.

7 Conway T, Heilman KM, Gopinath K, Peck K, Bauer R, Briggs RW *et al.* Neural substrates related to auditory working memory comparisons in dyslexia: an fMRI study. *J Int Neuropsychol Soc* 2008; **14**: 629–39.

8 Blau V, van Atteveldt N, Ekkebus M, Goebel R, Blomert L. Reduced neural integration of letters and speech sounds links phonological and reading deficits in adult dyslexia. *Curr Biol* 2009; **19**: 503–508.

9 Blau V, Reithler J, van Atteveldt N, Seitz J, Gerretsen P, Goebel R *et al.* Deviant processing of letters and speech sounds as proximate cause of reading failure: a functional magnetic resonance imaging study of dyslexic children. *Brain* 2010; **133**: 868–79.

10 Heim S, Grande M, Pape-Neumann J, van Ermingen M, Meffert E, Grabowska A *et al.* Interaction of phonological awareness and ‘magnocellular’ processing during normal and dyslexic reading: behavioural and fMRI investigations. *Dyslexia* 2010; **16**: 258–82.

11 Kast M, Bezzola L, Jäncke L, Meyer M. Multi- and unisensory decoding of words and nonwords result in differential brain responses in dyslexic and nondyslexic adults. *Brain Lang* 2011; **119**: 136–48.

12 Kovelman I, Norton ES, Christodoulou JA, Gaab N, Lieberman DA, Triantafyllou C *et al.* Brain basis of phonological awareness for spoken language in children and its disruption in dyslexia. *Cereb Cortex* 2012; **22**: 754–64.

13 Steinbrink C, Groth K, Lachmann T, Riecker A. Neural correlates of temporal auditory processing in developmental dyslexia during German vowel length discrimination: an fMRI study. *Brain Lang* 2012; **121**: 1–11.

14 Raschle NM, Stering PL, Meissner SN, Gaab N. Altered neuronal response during rapid auditory processing and its relation to phonological processing in prereading children at familial risk for dyslexia. *Cereb Cortex* 2014; **24**: 2489–501.

15 Shaywitz SE, Shaywitz BA, Pugh KR, Fulbright RK, Constable RT, Mencl WE *et al.* Functional disruption in the organization of the brain for reading in dyslexia. *Proc Natl Acad Sci U S A* 1998; **95**: 2636–2641.

16 Backes W, Vuurman E, Wennekes R, Spronk P, Wuisman M, van Engelshoven J *et al.* Atypical brain activation of reading processes in children with developmental dyslexia. *J Child Neurol* 2002; **17**: 867–71.

17 Shaywitz BA, Shaywitz SE, Pugh KR, Mencl WE, Fulbright RK, Skudlarski P *et al.* Disruption of posterior brain systems for reading in children with developmental dyslexia. *Biol Psychiatry* 2002; **52**: 101–110.

18 Siok WT, Niu Z, Jin Z, Perfetti CA, Tan LH. A structural-functional basis for dyslexia in the cortex of Chinese readers. *Proc Natl Acad Sci U S A* 2008; **105**: 5561–6.

19 Desroches AS, Cone NE, Bolger DJ, Bitan T, Burman DD, Booth JR. Children with reading difficulties show differences in brain regions associated with orthographic processing during spoken language processing. *Brain Res* 2010; **1356**: 73–84.

20 Díaz B, Hintz F, Kiebel SJ, von Kriegstein K. Dysfunction of the auditory thalamus in developmental dyslexia. *Proc Natl Acad Sci U S A* 2012; **109**: 13841–6.

21 Liu L, Wang W, You W, Li Y, Awati N, Zhao X *et al.* Similar alterations in brain function for phonological and semantic processing to visual characters in Chinese dyslexia. *Neuropsychologia* 2012; **50**: 2224–32.

22 Olulade OA, Gilger JW, Talavage TM, Hynd GW, McAteer CI. Beyond phonological processing deficits in adult dyslexics: atypical FMRI activation patterns for spatial problem solving. *Dev Neuropsychol* 2012; **37**: 617–35.

23 Peyrin C, Lallier M, Démonet JF, Pernet C, Baciu M, Le Bas JF *et al.* Neural dissociation of phonological and visual attention span disorders in developmental dyslexia: FMRI evidence from two case reports. *Brain Lang* 2012; **120**: 381–94.

24 Hernandez N, Andersson F, Edjlali M, Hommet C, Cottier JP, Destrieux C *et al.* Cerebral functional asymmetry and phonological performance in dyslexic adults. *Psychophysiology* 2013; **50**: 1226–38.

25 Kita Y, Yamamoto H, Oba K, Terasawa Y, Moriguchi Y, Uchiyama H *et al.* Altered brain activity for phonological manipulation in dyslexic Japanese children. *Brain* 2013; **136**: 3696–708.

26 van Ermingen-Marbach M, Pape-Neumann J, Grande M, Grabowska A, Heim S. Distinct neural signatures of cognitive subtypes of dyslexia: effects of lexicality during phonological processing. *Acta Neurobiol Exp (Wars)* 2013; **73**: 404–16.

27 Dole M, Meunier F, Hoen M. Functional correlates of the speech-in-noise perception impairment in dyslexia: an MRI study. *Neuropsychologia* 2014; **60**: 103–14.

28 Kronschnabel J, Brem S, Maurer U, Brandeis D. The level of audiovisual print-speech integration deficits in dyslexia. *Neuropsychologia* 2014; **62**: 245–61.

29 Dębska A, Łuniewska M, Chyl K, Banaszkiewicz A, Żelechowska A, Wypych M *et al.* Neural basis of phonological awareness in beginning readers with familial risk of dyslexia-Results from shallow orthography. *Neuroimage* 2016; **132**: 406–16.

30 Seki A, Koeda T, Sugihara S, Kamba M, Hirata Y, Ogawa T *et al.* A functional magnetic resonance imaging study during sentence reading in Japanese dyslexic children. *Brain Dev* 2001; **23**: 312–6.

31 Georgiewa P, Rzanny R, Gaser C, Gerhard UJ, Vieweg U, Freesmeyer D *et al.* Phonological processing in dyslexic children: a study combining functional imaging and event related potentials. *Neurosci Lett* 2002; **318**: 5–8.

32 Karni A, Morocz IA, Bitan T, Shaul S, Kushnir T, Breznitz Z. An fMRI study of the differential effects of word presentation rates (reading acceleration) on dyslexic readers’ brain activity patterns. *J Neurolinguistics* 2005; **18**: 197–219.

33 Brambati SM, Termine C, Ruffino M, Danna M, Lanzi G, Stella G *et al.* Neuropsychological deficits and neural dysfunction in familial dyslexia. *Brain Res* 2006; **1113**: 174–85.

34 Hoeft F, Meyler A, Hernandez A, Juel C, Taylor-Hill H, Martindale JL *et al.* Functional and morphometric brain dissociation between dyslexia and reading ability. *Proc Natl Acad Sci U S A* 2007; **104**: 4234–9.

35 Rimrodt SL, Clements-Stephens AM, Pugh KR, Courtney SM, Gaur P, Pekar JJ *et al.* Functional MRI of sentence comprehension in children with dyslexia: beyond word recognition. *Cereb Cortex* 2009; **19**: 402–13.

36 Wimmer H, Schurz M, Sturm D, Richlan F, Klackl J, Kronbichler M *et al.* A dual-route perspective on poor reading in a regular orthography: an fMRI study. *Cortex*; **46**: 1284–98.

37 Olulade OA, Flowers DL, Napoliello EM, Eden GF. Developmental differences for word processing in the ventral stream. *Brain Lang* 2013; **125**: 134–45.

38 Saralegui I, Ontañón JM, Fernandez-Ruanova B, Garcia-Zapirain B, Basterra A, Sanz-Arigita EJ. Reading networks in children with dyslexia compared to children with ocular motility disturbances revealed by fMRI. *Front Hum Neurosci* 2014; **8**: 936.

39 Olulade OA, Flowers DL, Napoliello EM, Eden GF. Dyslexic children lack word selectivity gradients in occipito-temporal and inferior frontal cortex. *NeuroImage Clin* 2015; **7**: 742–54.

40 Baillieux H, Vandervliet EJM, Manto M, Parizel PM, De Deyn PP, Mariën P. Developmental dyslexia and widespread activation across the cerebellar hemispheres. *Brain Lang* 2009; **108**: 122–32.

41 Eden GF, VanMeter JW, Rumsey JM, Maisog JM, Woods RP, Zeffiro TA. Abnormal processing of visual motion in dyslexia revealed by functional brain imaging. *Nature* 1996; **382**: 66–9.

42 Olulade OA, Napoliello EM, Eden GF. Abnormal visual motion processing is not a cause of dyslexia. *Neuron* 2013; **79**: 180–90.

43 Zhang Y, Whitfield-Gabrieli S, Christodoulou JA, Gabrieli JDE. Atypical balance between occipital and fronto-parietal activation for visual shape extraction in dyslexia. *PLoS One* 2013; **8**: e67331.

44 Diehl JJ, Frost SJ, Sherman G, Mencl WE, Kurian A, Molfese P *et al.* Neural correlates of language and non-language visuospatial processing in adolescents with reading disability. *Neuroimage* 2014; **101**: 653–66.

45 Beneventi H, Tønnessen FE, Ersland L. Dyslexic children show short-term memory deficits in phonological storage and serial rehearsal: an fMRI study. *Int J Neurosci* 2009; **119**: 2017–43.

46 Beneventi H, Tønnessen FE, Ersland L, Hugdahl K. Executive working memory processes in dyslexia: behavioral and fMRI evidence. *Scand J Psychol* 2010; **51**: 192–202.

47 Beneventi H, Tønnessen FE, Ersland L, Hugdahl K. Working memory deficit in dyslexia: behavioral and FMRI evidence. *Int J Neurosci* 2010; **120**: 51–9.

48 Wolf RC, Sambataro F, Lohr C, Steinbrink C, Martin C, Vasic N. Functional brain network abnormalities during verbal working memory performance in adolescents and young adults with dyslexia. *Neuropsychologia* 2010; **48**: 309–18.
